# Oncogene activated human breast luminal progenitors contribute basally located myoepithelial cells

**DOI:** 10.1186/s13058-024-01939-x

**Published:** 2024-12-18

**Authors:** Katharina Theresa Kohler, Jiyoung Kim, René Villadsen, Lone Rønnov-Jessen, Ole William Petersen

**Affiliations:** 1 Department of Cellular and Molecular Medicine, University of Copenhagen, Copenhagen, Denmark; 2 Section for Cell Biology and Physiology, Department of Biology, University of Copenhagen, Copenhagen, Denmark

**Keywords:** Human breast, Bipotent luminal progenitors, scRNA-seq, Breast cancer, Spatial mapping, Origin of the myoepithelial cells, PIK3CA^H1047R^, iHBEC^CD117^

## Abstract

**Background:**

Basal-like breast cancer originates in luminal progenitors, frequently with an altered PI3K pathway, and focally in close association with genetically altered myoepithelial cells at the site of tumor initiation. The exact trajectory behind this bi-lineage phenomenon remains poorly understood.

**Methods and results:**

Here we used a breast cancer relevant transduction protocol including hTERT, shp16, shp53, and PIK3CA^H1047R^ to immortalize FACS isolated luminal cells, and we identified a candidate multipotent progenitor. Specifically, we identified a keratin 23 (K23)^+^/ALDH1A3^+^/CALML5^−^ ductal-like progenitor with the potential to differentiate into CALML5^+^ lobular-like cells. We found that the apparent luminal phenotype of these oncogene transduced progenitors was metastable giving rise to basal-like cells dependent on culture conditions. In 3D organoid culture and upon transplantation to mice the bipotent progenitor cell line organized into a bi-layered acinus-like structure reminiscent of that of the normal breast gland.

**Conclusions:**

These findings provide proof of principle that progenitors within the human breast luminal epithelial compartment may serve as a source of correctly positioned myoepithelial cells. This may prove useful in assessing the role of myoepithelial cells in breast tumor progression.

**Supplementary Information:**

The online version contains supplementary material available at 10.1186/s13058-024-01939-x.

## Background

The human normal breast consists of a branched ductal-lobular parenchyma lined by an inner layer of luminal epithelial cells and an outer layer of myoepithelial cells [1]. In the course of breast tumor development the balance between these two major lineages is severely disturbed in favor of the luminal epithelial lineage (reviewed by [2]). In spite of the fact that most breast cancers originate exactly within the luminal epithelial lineage and that neighboring myoepithelial cells are considered tumor suppressive, transformation-induced lineage dynamics in this context remain understudied. Recent advances in single-cell transcriptomics and proteomics have revealed candidate normal breast cellular trajectories for the maintenance of tissue homeostasis and aberrations herein, potentially leading to breast cancer [3–7]. The human normal breast undergoes cyclic variation on a monthly basis, and even further variation in the course of pregnancy and lactation. This variation entails both cellular proliferation and involution. For such variation to be operational, the glandular tissue relies on a constantly fine-tuned balance between the two major epithelial lineages, and in glandular tissues of mice, heterotypic communication between luminal cells and basal cells has been reported to be essential for maintaining lineage fidelity [8]. In premalignant breast lesions, however, the luminal epithelial compartment becomes increasingly hyperplastic and dysplastic apparently without a concomitant increase in the myoepithelial compartment eventually to a point of carcinoma in situ [2]. One source of myoepithelial cells in such pre-malignant lesions are those of residual normal ducts into which the neoplastic cells have spread, but the finding of genetically abnormal myoepithelial cells in the area of tumor initiation has led to speculations of a bipotential progenitor in operation [9]. While some studies propose that myoepithelial cells are tumor suppressive and keep luminal epithelial cells, and in turn cancer cells, in check mechanically and by paracrine activity [10, 11], others suggest that the very presence of an intact layer of myoepithelial cells in carcinoma in situ predicts the risk of “invasive relapse” [12]. Remarkably, once overtly invasive, myoepithelial cells – also referred to as basal cells – presumably underlie and predict chemotherapy resistance in triple-negative breast cancer [13]. This is particularly intriguing since basal-like breast cancer arises in luminal progenitors [14, 15]. Therefore, at least in certain tumors, basal-like cells have a luminal origin. Moreover, it is well known that an oncogenic activation in mouse mammary gland as with PI3K, which is frequent in human breast cancer, leads to destabilisation of the luminal lineage (reviewed by [16]). Elucidation of the role of the myoepithelium is further complicated by the existence of different states of myoepithelial differentiation in normal breast including progenitors at the junction between lineages as observed by single cell RNA profiling [4, 5, 17]. Thus, single cell sequencing of human normal breast has revealed a small population of cells referred to as lineage primed intermediates [4]. Similarly, a cluster expressing both classical luminal and myoepithelial markers in addition to progenitor markers was found in both mouse mammary and human breast tissue and alluded to as potentially immature and bipotent [5]. Because of the apparent pleiotropic role of myoepithelial cells under normal and tumor conditions and the possible luminal origin of basal-like cells in human breast, a model of the proposed functional dynamics is highly warranted.

In the present study we describe the differentiation repertoire of a human breast epithelial progenitor cell line with respect to its ability to de novo generate myoepithelial-like cells. In a ground state culture platform based on a breast fibroblast feeder we observe correctly polarized luminal epithelial sphere formation and find de novo conversion into myoepithelial-like cells upon multi-lineage culture. By use of a fluorescent reporter under the K14 promoter at high spatiotemporal resolution we capture this conversion in the act. Finally, we show that in spite of the fact that progenitors are transduced with human telomerase reverse transcriptase (hTERT), short-hairpin(sh)RNAs targeting p16 and p53, and a pathologically relevant mutant form of Phosphatidylinositol-4,5-Bisphosphate 3-Kinase Catalytic Subunit Alpha (PIK3CA^H1047R^), these cells engage in bilayered acinar formation in an organoid assay and in vivo in mice. Together, our results demonstrate that breast progenitors even if mutated represent a potential source of functional myoepithelial tumor suppressors.

## Methods

### Human tissue

Normal breast tissue was received from 15 female donors undergoing reduction mammoplasty for cosmetic purposes. Material from the donated tissue has been included in previous studies. Approval for the use and storage of human tissue was obtained from the Scientific Ethical Committee of Region Hovedstaden (Reference H-2-2011-052). Tissue donations relied on informed consent, while donors remained anonymous except for their age at the time of surgery. Tissue for histology was snap-frozen in n-hexane or fixed in formaldehyde for paraffin embedding. Tissue for cell culturing was dissociated by opposing scalpels and further digested using 900 U/ml collagenase solution (Worthington Biochemical) overnight in Dulbecco's Modified Eagle Medium/Nutrient Mixture F-12 (DMEM/F-12, 1:1, Life Technologies) with 2 mM glutamine and 50 µg/ml gentamycin (Biological Industries). Primary breast organoids were isolated as described [18, 19] and stored in 90% fetal bovine serum (FBS, Sigma- Aldrich) and 10% dimethyl sulfoxide (Sigma-Aldrich) in liquid nitrogen until use. The biopsy used in this study to generate the progenitor cell lines, iHBEC^CD117^ and PIK3CA^H1047R^-iHBEC^CD117^, has been used previously as a source of a myoepithelial cell line [17].

### Fluorescence activated cell sorting (FACS)

Primary breast organoids were thawed and further dissociated using 0.25% trypsin in 100 mM EDTA (Sigma). Trypsin was inactivated with a few drops of FBS following resuspension of samples in HEPES buffer (Sigma) and filtering through a 100 µm filter. To isolate CD117^+^ luminal progenitors, cells were incubated with antibodies against the epithelial cell adhesion molecule (EpCAM) homolog, Trophoblast cell surface antigen 2 (TROP2, clone 162–46, brilliant violet (BV) 421/BV 510 conjugate, BD Biosciences, 563243/563244), low affinity nerve growth factor receptor (NGFR; also termed CD271, clone ME20.4, PE/APC conjugate, BioLegend, 345106/Cedarlane Laboratories, CL10013APC), hematopoietic growth factor receptor (c-kit, also termed CD117, clone 104D2, APC/PE conjugate, BD Biosciences, 333233/332785) and activated leukocyte cell adhesion molecule (ALCAM; also termed CD166, clone 3A6, AF488/BV421 conjugate, AbD SeroTec/BD, MCA1926A488/562936) for 45 min at 4 °C. As a control, cells were incubated in HEPES buffer without antibodies. After incubation, samples were spun down and washed twice with HEPES buffer followed by filtering through a 20 µm filter (BD) and addition of 1 µg/ml Fixable Viability Stain 780 (BD Horizon). Cells were analyzed and sorted by a multicolor compensated FACS-Aria™ Fusion Cytometer (BD) equipped with a 100 µm nozzle.

### Generation of luminal cell lines

To construct lentiviral particles, HEK293T cells were transfected using calcium phosphate method with packaging vectors containing pCMV-VSVG and pCMV-ΔR8.9 (gifts from Frederik Vilhardt, University of Copenhagen) and lentiviral vectors containing pLenti-shp16-hygro (gift from Eric Campaue, Addgene plasmid #22264), pLV-hTERT-IRES-Hygro (gift from Tobias Meyer, Addgene plasmid #85140) [20], pLVUH-shp53-eGFP (gift from Patrick Aebischer & Didier Trono, Addgene plasmid #11653) [21] and pHAGE-PIK3CA-H1047R-puro (gift from Gordon Mills & Kenneth Scott, Addgene plasmid #116500) [22]. 48 h after transfection, supernatants were collected, filtered and concentrated by centrifugation. Luminal progenitor cells were transduced by culturing with viral supernatants for three days followed by selection with hygromycin (67 µg/ml) and puromycin (0.67 µg/ml) or by FACS based sorting of GFP positive cells. Concurrent transduction with hTERT and shp16 was applied in a primary culture of TROP2^+^/CD271^−^/CD117^+^ cells to generate the immortalized human breast luminal progenitor cell line (iHBEC^CD117^). Subsequently, iHBEC^CD117^ was transduced with shp53 in second passage upon selection. GFP-expressing shp53 transduced cells were sorted by FACS, and expanded for two passages prior to transduction with PIK3CA^H1047R^ in passage four. This cell line is hereafter referred to as PIK3CA^H1047R^-iHBEC^CD117^. This serial transduction method has previously been successfully applied in a primary culture of TROP2^+^/CD271^+^ myoepithelial cells [17]. iHBEC^CD117^ and PIK3CA^H1047R^-iHBEC^CD117^ cells have been used for experiments in a range between passage 9 and 24. We also employed a previously established luminal cell line iHBEC^ERpos^, representing mature luminal/hormone sensing cells immortalized by hTERT/shp16, which was originally sorted from primary culture as EpCAM^+^/CD271^−^/CD166^high^ cells [23–25]. In order to generate PIK3CA^H1047R^-iHBEC^ERpos^ cells were also sequentially transduced with shp53 and PIK3CA ^H1047R^. All cell lines were further expanded by a weekly split ratio of approximately 1:6. For generation of PIK3CA^H1047R^-iHBEC^CD117^ single cell derived clones, FACS was used as described above with antibodies against TROP2 and CD271. TROP2^+^/CD271^−^ cells were sorted as single cells in wells of 96-well plates (Corning, 353872) and expanded. Clones were analyzed by FACS and immunostaining and selected clones (including 3D9 and 2D10) were used for subsequent experiments.

### Cell culture

All luminal cells and cell lines were maintained in Primaria flasks (Corning, 353,808) in TGFβR2i-1 medium, supporting multi-lineage propagation of breast epithelial cell progenitors [23, 24]. This medium is based on DMEM with high glucose and no calcium/F-12 (3:1, Life Technologies) supplemented with 5 nM amphiregulin (Peprotech), 5 µg/ml insulin (Sigma Aldrich), 1.8 × 10^–4^ M adenine (Sigma-Aldrich), 10 μM Y-27632 (Axon Medchem) and 5% FBS, with the addition of inhibitors of TGF-β receptor signaling, SB431542 (10 μM, Axon Medchem) and RepSox (25 μM, Sigma-Aldrich) [23, 24].

For ground state culture we used another condition previously developed to support lineage restriction of luminal epithelial cells as acini, and myoepithelial cells as myodifferentiated cells, respectively [17, 24–26]. In this condition, a fibroblast feeder is indispensable [27, 28]. The ground state culture medium is based on DMEM/F-12, 1:1, supplemented with 2 mM glutamine, 1 μg/ml hydrocortisone (Sigma-Aldrich), 9 μg/ml insulin, 5 μg/ml transferrin (Sigma-Aldrich), 100 μM ethanolamine (Sigma-Aldrich), 20 ng/ml basic fibroblast growth factor (PeproTech), 5 nM amphiregulin, 10 μM Y-27632, 180 μM adenine, 20 μl/ml serum replacement B27 (Life Technologies), 25 μM Repsox, and 10 μM SB431542 as previously described (BBMYAB/ Myo medium; [17, 24, 26]). The fibroblast feeder includes human breast endo-glin (ENG, also termed CD105)^high^/dipeptidyl peptidase-4 (DPP4, also termed CD26)^low^ intralobular fibroblasts (passage 12–15, [27]) routinely cultured in DMEM/F-12 supplemented with 5% FBS and 2 mM glutamine on collagen-coated flasks (Nunc, 8 μg/cm^2^, PureCol, Cell Systems). All cultures were kept at 37 °C in an incubator providing a humidified 5% CO_2_ atmosphere. All cell lines have been routinely tested for mycoplasma contamination and found negative.

### 3D organoid culture

To form organoids, 10,000 PIK3CA^H1047R^-iHBEC^CD117^ cells were co-suspended with 2,500 normal breast fibroblasts (passage 12–15) or functionally equivalent myofibroblast-like cancer associated fibroblasts (myCAFs) [28, 29] in 100 µl of a mixture of 1:1 growth factor reduced Matrigel™ (Corning) and collagen I (Advanced Biomatrix) and carefully pipetted into a well of a 24-well plate (Thermo Scientific) as a drop, in triplicates. After solidifying the gel at 37 °C, 500 µl of Mammary Epithelial Cell Growth Medium (MEGM™, Lonza,) supplemented with 20 ng/ml epidermal growth factor (PeproTech), 4 μg/ml heparin (Sigma-Aldrich), 20 ng/ml basic fibroblast growth factor, 20 μl/ml B27, 500 nM A83-01 (Tocris), 25 μM Repsox and 10 µM SB431542 was added, and gels were incubated for 2–4 weeks. Gels were snap-frozen in n-hexane (Sigma-Aldrich) and stored at -80 °C.

### Mouse xenografts

Xenotransplantation was conducted with approval by the Animal Experiments Inspectorate (2022-15-0201-01248). To humanize the microenvironment and support the unfolding of the epithelial cells´ differentiation potential, for each injection, one million luminal progenitor cells (iHBEC^CD117^cells, PIK3CA^H1047R^-iHBEC^CD117^cells or clones of PIK3CA^H1047R^-iHBEC^CD117^cells) or hormone sensing cells (iHBEC^ERpos^cells or PIK3CA^H1047R^-iHBEC^ERpos^cells) were mixed with 250,000 normal breast fibroblasts (passage 12–16) and resuspended in a 1:1 Matrigel and collagen mixture as previously described [17, 26]. Cell suspensions were injected subcutaneously in NOD.Cg-*Prkdc*^*SCID*^*Il2rg*^*tm1Wjl*^/SzJ (NOG) mice (Taconic), in the region of the fourth mammary fat pads (n = 12 injections for PIK3CA^H1047R^-iHBEC^CD117^, passage 9–24, n = 4 per each of its clones, n = 6 for iHBEC^CD117^, n = 6 for iHBEC^ERpos^, and n = 12 for PIK3CA^H1047R^-iHBEC^ERpos^). 0.67 µg/ml 17β-estradiol (E2, Sigma-Aldrich) was supplied through the drinking water. After two months, the mice were sacrificed, and gels within mammary glands were excised and snap-frozen in n-hexane for cryosectioning or fixed in 10% formalin (CellPath) for paraffin embedding.

### Generation of K19 and K14 reporter PIK3CA^H1047R^-iHBEC ^CD117^ cell lines

A glycine/serine (GS) linker-miRFP670-SV40 poly (A) tail sequence was introduced in front of the stop codon of the *KRT19* genomic sequence in PIK3CA^H1047R^-iHBEC^CD117^ cells to trace the luminal lineage in real-time. For this site-specific knock-in, Clustered Regularly Interspaced Short Palindromic Repeats (CRISPR)/CRISPR associated protein 9 (Cas9) induced a double strand break in one allele of *KRT19*, and the donor sequence was recombined into the locus by homology directed repair. Two single guide (sg)RNAs (GCUGCCUCAGAGGACCUUGG and CAACAAUUUGUCUG CCUCCA) targeting the stop codon region of *KRT19* were designed using a sgRNA design tool (Synthego, V1.3). The donor sequence included a GS linker and miRFP670 flanked by 500 base pairs of homology arms according to the *KRT19* genomic DNA at insert site. A silent point mutation was introduced to the protospacer adjacent motifs (PAM) sequence, and the donor sequence was cloned into the pUC57-Amp vector. Cloning was conducted by Synbio Technologies. 18 h prior to electroporation, PIK3CA^H1047R^-iHBEC^CD117^ cells were treated with 100 ng/ml nocodazole (Sigma-Aldrich) for synchronizing the cell cycle in G2/M phase to enhance knock-in efficiency. After linearization and amplification using PCR, 1 µg donor template was co-transfected with nuclear localizing Cas9 protein (spCas9-NLS, Synthego) and sgRNAs (9:1 with Cas9) into PIK3CA^H1047R^-iHBEC^CD117^ cells using electroporation according to the manufacturer's instructions with the Amaxa® HMEC Nucleofector® Kit (W-001 program, Lonza). After transfection, cells were cultured and expanded. To generate the PIK3CA^H1047R^-iHBEC^CD117^ K19 reporter cell line derived by a single cell clone, single cells were sorted by FACS as described above with the gate of TROP2^+^/CD271^−^ and miRFP670^+^ into Primaria 96-wells (Corning, 353872). After expansion, genomic DNA was extracted from clones using the DNeasy Blood & Tissue Kit (Qiagen) and clones were analyzed for correct integration by Sanger sequencing (Eurofins Genomics). Further validation was performed by FACS analysis and immunostaining for co-localization of miRFP670 and K19. The near-infrared fluorescent miRFP670 protein was excited by a violet laser and K19 fluorescence was detected as described.

Subsequently, to construct a K14 reporter cell line on the K19 reporter background, a lentiviral vector containing 2000 bp *KRT14* promoter fused with RFP was purchased (System Biosciences) and used for constructing lentiviral particles applying the same method mentioned above. Viral particles were transduced into PIK3CA^H1047R^-iHBEC^CD117^ K19 reporter cells.

### Bulk RNA sequencing

PIK3CA^H1047R^-iHBEC^CD117^ cells were sorted by FACS as described above using antibodies against TROP2 and CD271. TROP2^+^/CD271^−^ and TROP2^+^/CD271^+^ cells were lysed separately using TRIzol™ Reagent (Invitrogen) by incubating cells with TRIzol for 10 min. RNA was isolated using the Direct-zol RNA Microprep kit (Zymo Research) following the manufacturer’s instructions. Sequencing was conducted by BGI tech solutions and reads were aligned to human genome Homo_sapiens_NCBI_GCF_000001405.39_GRCh38.p13. DESeq2 method [30] was used to determine differentially expressed genes (n = 2 per group). R (versions 4.3.0 – 4.4.0) was used for further analysis and construction of graphs.

### Single cell RNA Sequencing (scRNA-seq) and bioinformatics

scRNA-seq was conducted with PIK3CA^H1047R^-iHBEC^CD117^ cells at the Genomics Platform (part of the Novo Nordisk Foundation Center for Stem Cell Medicine, reNEW Copenhagen). 10X Chromium Next GEM single cell 3’ kit v3.1 (PN-1000268) was used according to the manufacturer’s instructions for sc capture, reverse transcription and cDNA amplification as well as library preparation. Sequencing was conducted with a NextSeq2000 using a P2-100 kit.

Data were demultiplexed and a count matrix was generated using Cell Ranger. The count matrix was read into R and processed using “Seurat” package (v5.0.3, [31]). Cells with a mitochondrial read of more than 10% were excluded as well as cells with a gene count below 2000. After filtering, 2756 cells remained for downstream analysis. Data were normalized and PCA was run with 2000 most variable expressed genes. Finally, clusters were defined using Seurat default settings in conjunction with Louvain algorithm, except for setting the resolution at 0.5. For visualization, Uniform Approximation and Projection (UMAP) dimensionality reduction technique was used with 12 principal components as input. After analyzing differentially expressed genes, two of the clusters, were combined to one (cluster 0) according to their very similar expression of genes.

A diffusion map, a method for non-linear dimension reduction, was employed to reveal the underlying structure and differentiation hierarchy within the dataset [32]. This analysis was conducted using the ‘destiny’ package in R (v3.18.0, [33, 34]) with default settings for “DiffusionMap” function. Concurrently, a pseudotime trajectory analysis was conducted, resulting in a diffusion pseudotime (DPT) metric [35]. DPT is a measure based on the transition probability of a diffusion process, allowing us to order individual cells according to their progression through a biological process.

ScRNA-seq data for primary tissue were generated as previously described [7, 17]. Data for luminal and myo-epithelial cells were combined, and UMAP dimensionality reduction was run using previously defined lineage-specific markers as well as genes that allow to distinguish cells of spatial origins in breast tissue.

### Live cell imaging

PIK3CA^H1047R^-iHBEC^CD117^ cells with fluorescence reporters were cultured in ground state culture conditions in µ-Slide 8 well^high^ Grid-500 wells (ibidi). Images were acquired one week after seeding and a second set of images was acquired after an additional week in multi-lineage culture conditions. The CellObserver spinning disc microscope (Zeiss) was provided by the Core Facility for Integrated Microscopy (CFIM, University of Copenhagen) and used with a 20 × Magnification/0.8 NA objective for imaging. Tile images were acquired to cover the whole area of the grid in each well. Images of the same wells between different time points were acquired, and regions of interest were compared.

### Immunostaining

Cryostat sections, FACS-derived cell smears and PIK3CA^H1047R^-iHBEC^CD117^ cell cultures were fixed and stained as previously described [7, 17]. ProLong™ Gold antifade reagent with DAPI (Invitrogen) was applied to fluorescent staining. Paraffin-embedded sections were deparaffinized and processed according to standard protocols. Heat mediated antigen retrieval was performed in either citrate buffer at pH 6 or Tris-Ethylene Glycol-bis (2-aminoethylether)-N,N,N′,N′-Tetraacetic Acid (EGTA) buffer at pH 9, depending on antibodies (Table 1). After a blocking step for 30 min with 10% goat serum at room temperature (RT) sections were incubated overnight at 4 °C with primary antibodies diluted in 10% goat serum followed by 30 min incubation with secondary antibodies at RT. Images were acquired with a Zeiss LSM700 confocal microscope or a Leica DM5500B with a DFC550 camera.

**Table 1:** Antibody list

Antigen	Clone	Company	Catalogue nr	Dilution; method
ALDH1A3	GT926	GeneTex	GTX633822	1:100; P, pH 6/9
aSMA	1A4	Sigma-Aldrich	A2547	1:500; C
CALML5	2F10	Abnova	H00051806-M16	1:50; P, pH 9
CALML5	119	Invitrogen	MA529080	1:400; P, pH 6
CAM5.2	CAM5.2	Becton Dickinson/BD Biosciences	345,779	1:50; CS
K14	LL002	Nordic BioSite	MonX10687	1:300; C/ CS/CC
K19	BA16	Abcam	Ab20210	1:300; C/CC
K17	E3	Dako	M7046	1:100; CS
K23	–	Cloud-Clone Corp	PAP902Hu01	1:2000; P, pH 9
MUC1 (MAM-6)	115D8	Monosan	MON9005-1	1:10, C/CC
OLFM4	–	Atlas antibodies	HPA077718	1:1000; C
P63	7Jul	Novocastra	NCL-L-p63	1:50; C

C = cryo, P = paraffin, CS = cell smear, CC = cell culture, pH 6/ pH 9 = pre-treatment in buffer at pH 6 or pH 9

### Statistical analysis

Statistical analysis was performed using either R Studio (version 4.3.0 – 4.4.0) or GraphPad Prism (version 9.0.0). For each experiment statistical tests were chosen independently and tests were specified in addition to significance in the figure legends. Significance is indicated as follows: p < 0.05 (*), p < 0.01 (**), p < 0.005 (***), and p < 0.001 (****).

## Results

### Generation and validation of transduced human breast progenitor-derived cells with clinically relevant driver mutations

**Fig. 1:**
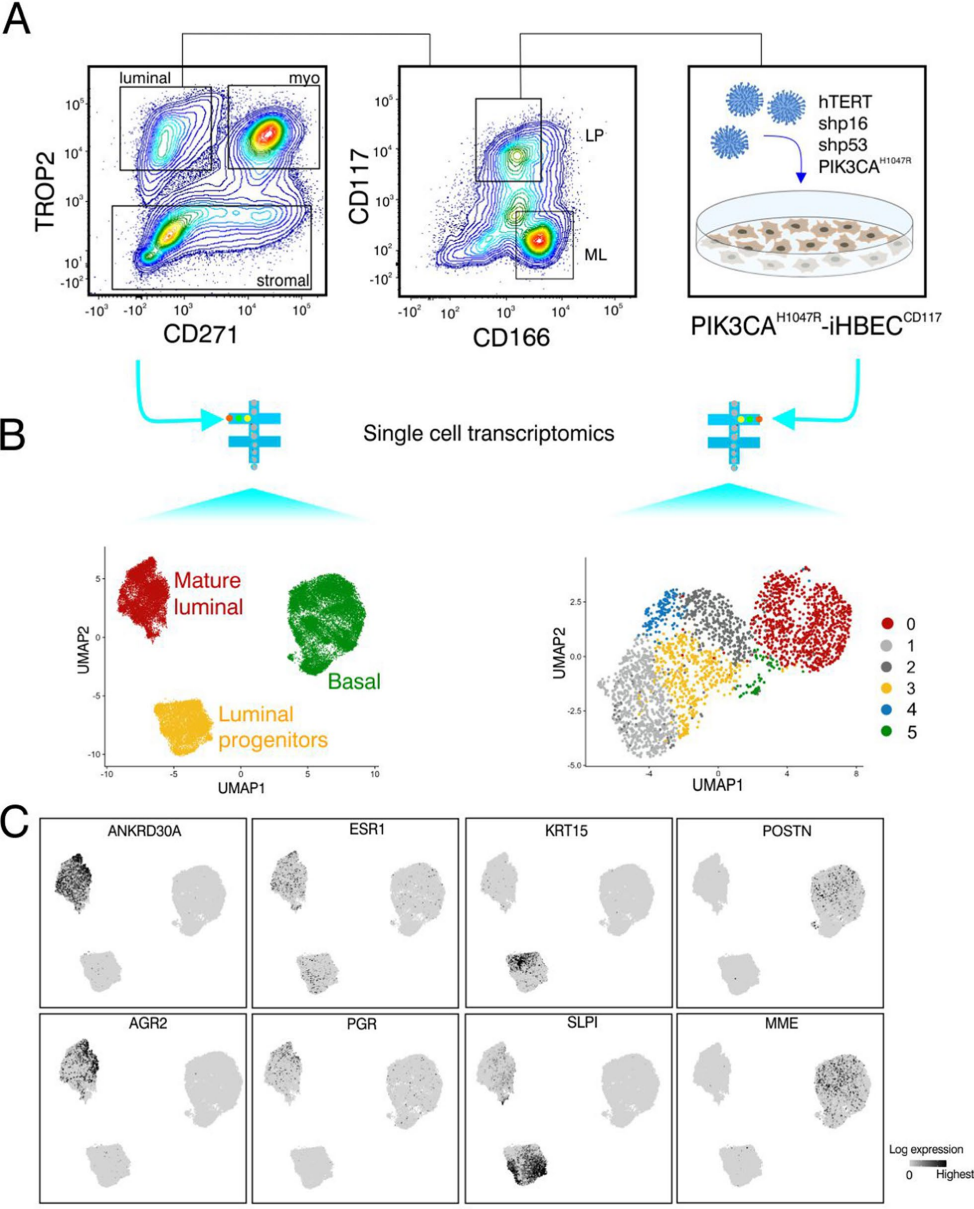
Isolation, immortalization and characterization of luminal progenitors. **A** FACS diagrams showing the gating strategy for sorting of luminal progenitors. Uncultured single cells were harvested from freshly thawed collagenase digests and further submitted to a TROP2/CD271/CD117/CD166 FACS protocol to separate luminal, myoepithelial and stromal cells (left) and further separate luminal progenitor (LP) from mature luminal (ML) cells (middle). Candidate TROP2^+^/CD271^−^/CD117^+^/CD166^−^ progenitors were gated as illustrated with squares and plated in culture for sequential transduction with hTERT, shp16, shp53 and PIK3CA^H1047R^ (right). The resulting cell line is referred to as PIK3CA^H1047R^-iHBEC^CD117^. **B** scRNA sequenced basal and luminal cells were resolved by UMAP dimensionality reduction and clustering of uncultured cells from three reduction mammoplasty samples (left, n = 3) and compared with data from the PIK3CA^H1047R^-iHBEC^CD117^ cell line (right). Clusters with related expression profiles were annotated with identical colors as shown on the left (red for ML, yellow for LP, and green for Basal). PIK3CA^H1047R^-iHBEC^CD117^ clusters with no equivalent among uncultured cells were colored gray and cluster 4 with a stem like profile was colored blue. **C** Examples of genes enriched in ML (*ANKRD30A*, *AGR2*, *ESR1* and *PGR*), LP (*KRT15* and *SLPI*) and Basal (*POSTN* and *MME*) clusters, respectively

TROP2 in combination with CD271 and CD166 in combination with CD117 are cell surface proteins that can be used to isolate human breast epithelial cells [17, 23]. In uncultured cells from reduction mammoplasties, the TROP2/CD271 combination in itself effectively separates uncultured luminal epithelial cells and myoepithelial cells [7, 17, 36] as confirmed here by staining for K7/8, K14 and K17 of smeared cells (Fig. 1A and Additional file 1: Fig. S1), while CD117/CD166 distinguishes mature luminal cells from candidate progenitor cells (Fig. 1A). This is consistent with our previous data showing that the majority of primary myoepithelial cells stain positive for K14 and K17 [17, 23], while primary luminal cells are positive for K7/K8 [26, 37]. To reflect the most frequent driver mutations in human breast cancer﻿ [38], the luminal progenitors were first immortalized by viral transduction of hTERT and shp16 (here referred to as iHBEC^CD117^) and then sequentially transduced with shp53 and the PIK3CA^H1047R^ oncogene (Fig. 1A) - a combination previously employed to establish myoepithelial cell lines [17]. Notably, introducing PI3KCA^H1047R^ alone induces cellular senescence in mammary epithelial cells [39]. The resulting cell line, PIK3CA^H1047R^-iHBEC^CD117^, maintained the luminal lineage as luminal keratins (K7/K8) were expressed in all cells. Myoepithelial keratin K14, on the other hand, was mainly expressed in CD271^+^ cells as shown by staining of smears from the CD271^+^ gate of a TROP2/CD271 FACS protocol (41.0 ± 18.7% of TROP2^+^/CD271^+^ cells compared 2.9 ± 0.9% of TROP2^+^/CD271^−^ cells).

**Fig. 2:**
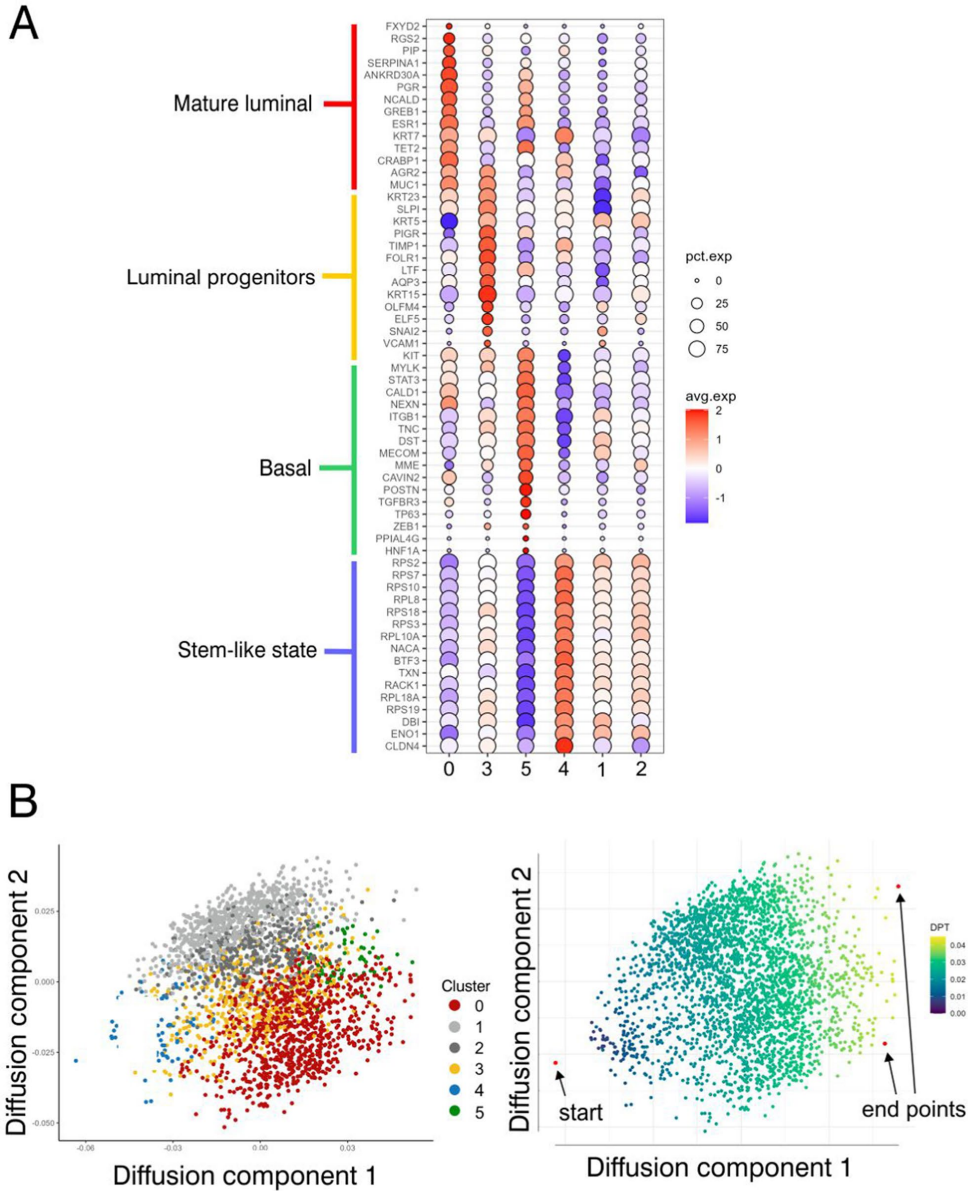
Inferring cell-types and potency states in PIK3CA^H1047R^-iHBEC^CD117^ based on scRNA**-**seq.** A** Bubble plot showing the average normalized gene expression in PIK3CA^H1047R^-iHBEC^CD117^ and the percentage of cells expressing lineage-related genes that distinguish clusters of epithelial cell states. Each column corresponds to a specific cell cluster and the rows correspond to a list of key marker genes. Colored lines on the left side of the plot indicate the cell type that these genes mark. Note that clusters 1 and 2 appear as if they represent intermediate states with no obvious equivalent in the normal human breast. **B** Diffusion map of PIK3CA^H1047R^-iHBEC^CD117^ cells colored according to the original single-cell clustering (left). Same diffusion map colored according to pseudotime, inferring a root state (start in left) at the apex of the luminal progenitor (corresponding to cluster 4) and two terminally differentiated states (end points in right) (right). The color bar value indicates the differentiation process as referred to diffusion pseudotime (DPT)

The identity of PIK3CA^H1047R^-iHBEC^CD117^ in terms of lineages and cell states within the human breast epithelium was further evaluated by scRNA-seq. 38,964 uncultured, sorted cells from three independent donors who underwent reduction mammoplasty served as a reference dataset. This dataset encompasses luminal and myoepithelial cells, which have previously been published independently [7, 17]. Clustering of sequencing data from normal breast has previously identified three clusters representing one basal and two luminal cell populations [3, 4, 40], also referred to by others as basal, secretory and hormone responsive cells, here termed basal cells, luminal progenitors and mature luminal cells [4, 41, 42]. Indeed, we here defined three cell populations which showed distinct expression of known markers defining the basal, luminal progenitor and mature luminal cells, respectively (Fig. 1B, left). Clustering of 2,756 PIK3CA^H1047R^-iHBEC^CD117^ cells revealed six clusters out of which three had gene expressions in common with the clusters in the reference dataset (Fig. 1B, right). Comprehensive analysis in expression of typical breast lineage marker genes has been shown previously [7, 17]. Among them, indeed, mature luminal makers *ESR1, PGR, ANKRD30A*, and *AGR2*, luminal progenitor markers *KRT15* and *SLPI,* and basal markers *POSTN and MME*, were found in each of the respective annotated clusters of PIK3CA^H1047R^-iHBEC^CD117^ (Fig. 2A) projected onto the basal, luminal progenitor, and mature luminal patterns of uncultured cells (Fig. 1C). *ESR1, PGR, ANKRD30A,* and *AGR2* as well as *KRT15* and *SLPI* have been previously defined as markers that distinguish mature luminal from luminal progenitor cells, respectively, in scRNA-seq data [7, 40, 43, 44]. Periostin (*POSTN*), a ligand of αvβ3 integrins and *MME*, encoding membrane metalloendopeptidase (also termed CD10) have been extensively used as myoepithelial markers [45–47]. To substantiate the iterative subclustering of PIK3CA^H1047R^-iHBEC^CD117^ cells and further resolve their lineage- and cell state identity we searched for canonical markers within existing datasets [3–5, 41–43, 48–50]. The result of this search is summarized in a bubble plot format in Fig. 2A. Clearly, even if incomplete, the differentiation repertoire of PIK3CA^H1047R^-iHBEC^CD117^ was compatible with that of the epithelial compartment of the human breast. In addition, we found that cluster 4 exhibited a profile rich in ribosomal genes and *CLDN4* (Fig. 1B, right and Fig. 2A). These features have been characterized by others as stem cell- or progenitor-like [3, 40, 44]. The finding of a ribosomal protein profile along with the nitric oxide response transcription factors *BTF3* and *TXN* in a subset of cells would be in accordance with the presence of bi-potent progenitors [3]. Potential lineage trajectories were further examined by diffusion map algorithm, which positioned cluster 4 at the apex of the differentiation trajectory (Fig. 2B). Based on these results, PIK3CA^H1047R^-iHBEC^CD117^appears to exhibit prominent cellular diversity, including progenitors and mature differentiation states.

### Derivatives of luminal progenitors of ductal origin populate the entire ductal-lobular tree

**Fig. 3:**
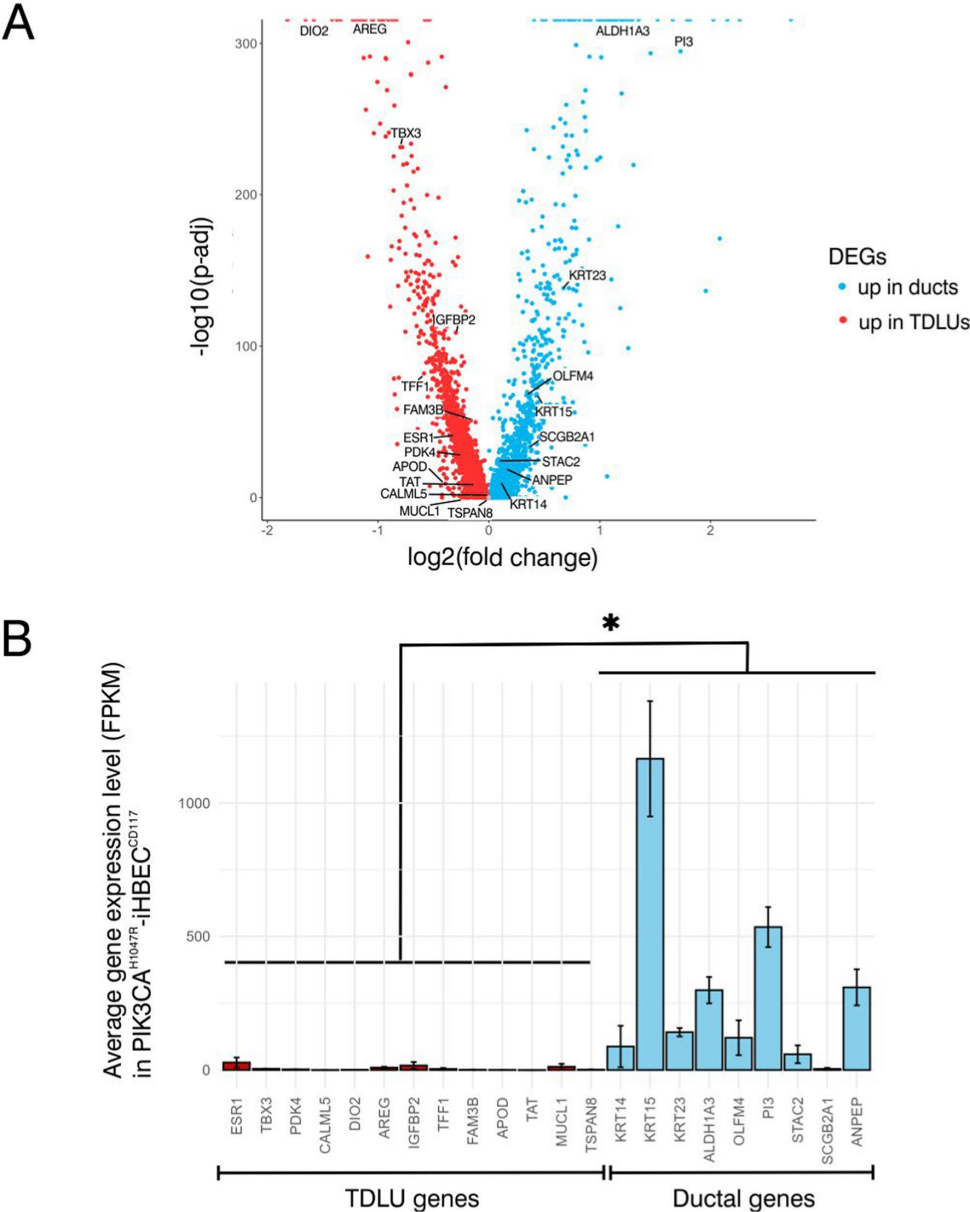
Profiling reveals that PIK3CA^H1047R^-iHBEC^CD117^ preferentially corresponds to ductal properties. **A** Volcano plot showing differentially expressed genes between ductal and TDLU-located luminal cells in normal human breast tissue. Genes expressed at a higher level in ducts are indicated in blue, while red color denotes genes higher expressed in TDLUs. Thirteen lobular genes and nine ductal genes were selected based on their correlation with ML and LP states, respectively. **B** Bar plot showing average gene expression levels (FPKM) of ductal and lobular genes in PIK3CA^H1047R^-iHBEC^CD117^ from bulk RNA-seq, showing higher expression of ductal markers. Error bars indicate standard deviation of mean. *p < 0.05, tested by two-tailed t test (n = 4)

**Fig. 4:**
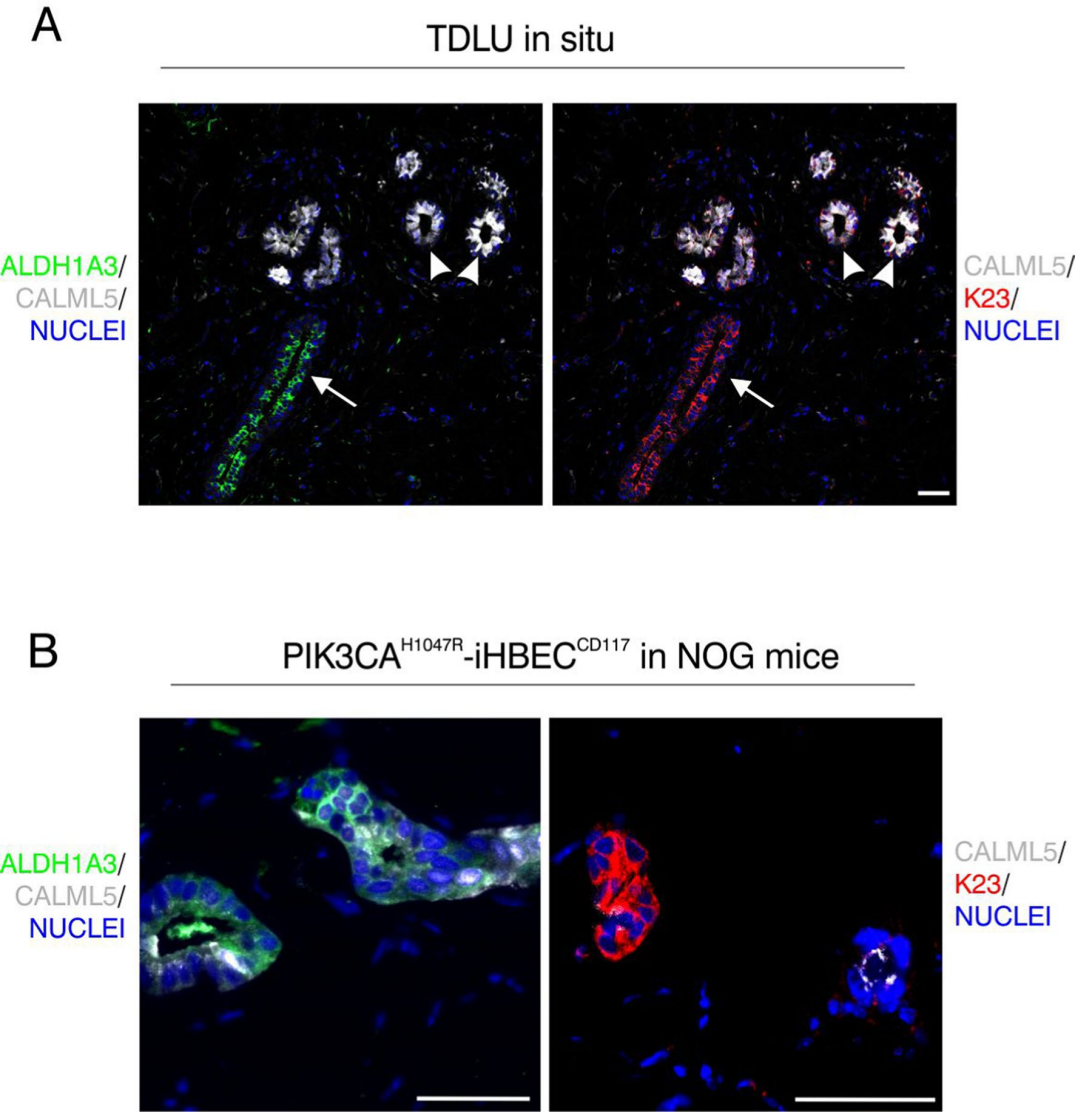
PIK3CA^H1047R^-iHBEC^CD117^ luminal progenitors give rise to both lobular and ductal luminal cells in vivo. **A** Confocal imaging of a representative paraffin section of reduction mammoplasty specimens multicolor-stained for ALDH1A3 (green), CALML5 (white) and K23 (red), and visualized to enhance a combination of white and green (left) and white and red (right), respectively. CALML5 is primarily confined to acini (9 out of 9 biopsies), while ALDH1A3 and K23 are mostly found in extralobular terminal ducts (ETD) (8 out of 10 and 5 out of 9 biopsies, respectively). Arrowheads = acini, arrows = ETDs. Scale bar = 50 µm. **B** Confocal imaging of paraffin sections of PIK3CA^H1047R^-iHBEC^CD117^ cells in NOG mice costained for ALDH1A3 (green) and CALML5 (white; left), or keratin K23 (red) and CALML5 (white; right). Note that PIK3CA^H1047R^-iHBEC^CD117^ expresses both ductal and lobular markers, albeit with a tendency of separation. Scale bar = 50 µm

To investigate whether PIK3CA^H1047R^-iHBEC^CD117^ faithfully represents cells in situ, we next sought to map the bipotent progenitor profile found in culture to the ductal-lobular tree of the tissue of origin. Previous observations by us and others have roughly resolved progenitor marker expression into that of ducts/terminal ducts and acini [26, 44]. Integrating our previously generated scRNA-seq profiles from FACS-isolated luminal cells of micro-collected ducts and terminal duct lobular units (TDLUs) [7] with that of progenitor- and mature breast cell cluster accessibility datasets [43] allowed us to assign 22 genes of luminal progenitor- or mature luminal epithelial cells preferentially to ducts and TDLUs, respectively (Fig. 3A). Interestingly, genes of mature luminal clusters, including *TBX3*, *PDK4*, *FAM3B*, *MUCL1*, and *AREG*, confirmed accumulation exclusively in the TDLU profile of the primary luminal cells (Fig. 3A). While genes higher expressed in TDLUs exhibited low expression in PIK3CA^H1047R^- iHBEC^CD117^, expression of ductal progenitor markers was significantly higher as shown in Fig. 3B. Among the 22 genes identified, we were able to obtain antibodies and optimize multi color imaging for five of them: Calmodulin like 5 (*CALML5*), K15 (*KRT15*), K23 (*KRT23*), Aldehyde Dehydrogenase 1A3 (*ALDH1A3*), and Olfactomedin 4 (*OLFM4*). We previously described K15 as a progenitor marker mainly seen in luminal epithelial cells of the ducts and terminal ducts [7]. Here, consistent with the mRNA expression data, we found that CALML5 was restricted to acini, and as such to our knowledge represents the first marker exclusively expressed by lobular luminal cells (Fig. 4A). In contrary, ALDH1A3, K23 and OLFM4 were preferentially expressed in ducts and terminal ducts in situ (Fig. 4A and Additional file 1: Fig. S2A). Notably, in organoid cultures, PIK3CA^H1047R^-iHBEC^CD117^ maintained its ductal origin by showing strong expression of K23 and OLFM4 with the absence of CAML5 staining (Additional file 1: Fig. S3A, B). When transplanted to mice, however, PIK3CA^H1047R^-iHBEC^CD117^ cells expressed not only ductal markers OLFM4, K23 and ALDH1A3, but also expressed CALML5 in subsets of cells often different from those expressing ALDH1A3 and K23 (Fig. 4B and Additional file 1: Supplementary Fig. S2B). This suggests that luminal progenitors, albeit ductal in origin, have the potential to differentiate into both lobular and ductal luminal cells.

### PIK3CA^H1047R^-iHBEC^CD117^ contribute a subset of cells that differentiate into the myoepithelial lineage

**Fig. 5:**
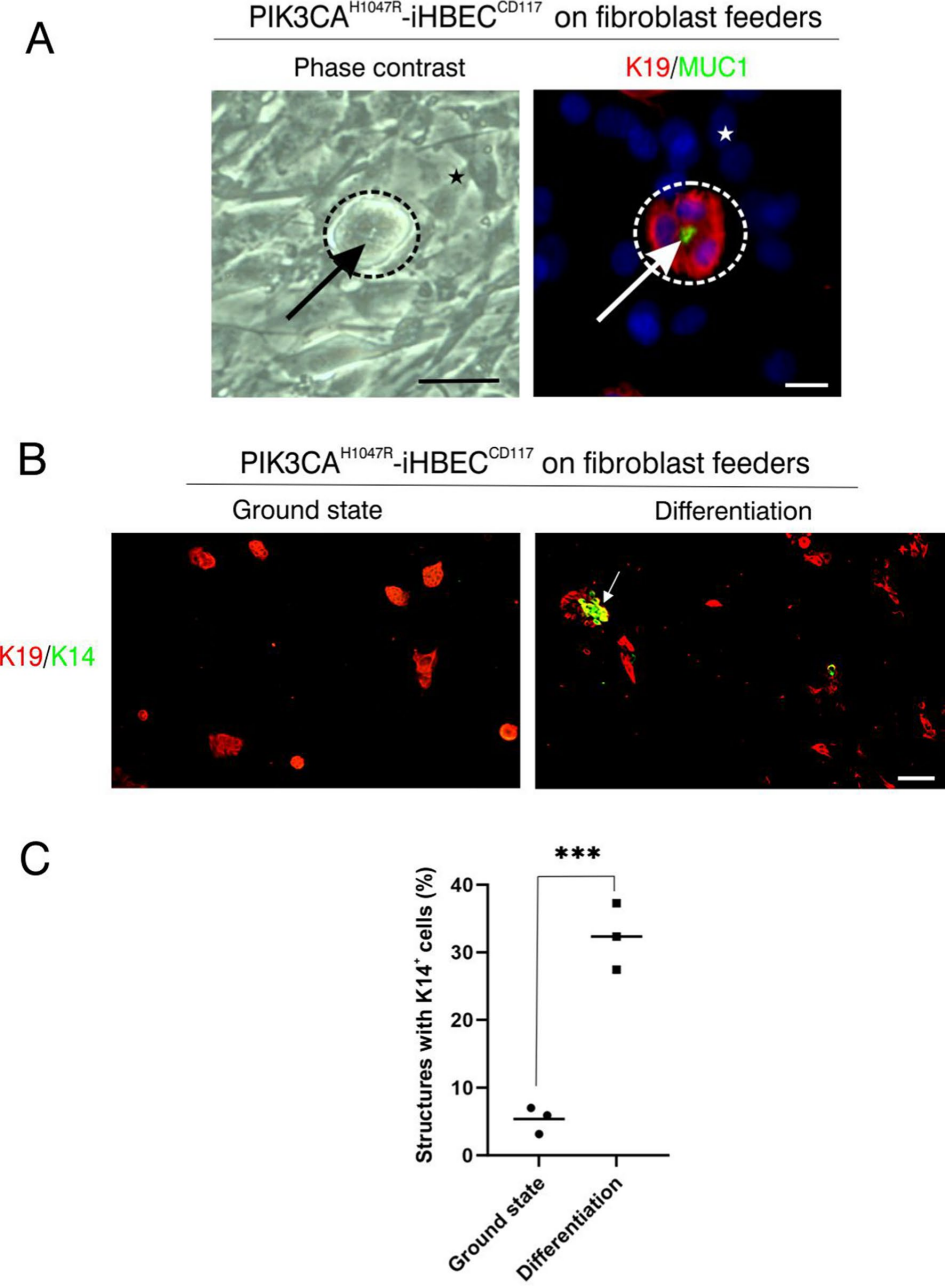
A subset of PIK3CA^H1047R^-iHBEC^CD117^ differentiate into the myoepithelial lineage. **A** High magnification images of PIK3CA^H1047R^-iHBEC^CD117^ in ground state culture on fibroblast feeders. Phase contrast image (left) shows a sphere (indicated by dotted line) with lumen (arrow), and immunostaining (right) for K19 (red) and MUC1 (green) and nuclei stained with DAPI (blue) reveals polarity by apical expression of MUC1. Stars denote fibroblasts. Scale bar = 50 µm (left) and 25 µm (right). **B** Multicolor imaging of PIK3CA^H1047R^-iHBEC^CD117^ stained for K19 (red) and K14 (green) in ground state conditions (left) and after 72 h of differentiation in multi-lineage conditions (right). Note that acinus-like K19^+^/K14^−^ luminal structures form in ground state culture, and that K14^+^ cells (arrow) frequently appear after differentiation. Scale bar = 100 µm. **C** Dot plot with the relative number (%) of acinus-like structures associated with K14^+^ cells in ground state and differentiation conditions, respectively. There are significantly more structures with associated K14^+^ cells after differentiation (*** p < 0.001, tested by two-tailed t test, n = 3). Bars indicate the average of three experiments

We next assessed the functional activities of PIK3CA^H1047R^-iHBEC^CD117^ by submitting it to culture conditions known to influence lineage commitment. Thus, cells were first exposed to a culture condition defined as a ground state [17, 24]. This revealed a clear propensity of PIK3CA^H1047R^-iHBEC^CD117^ to rest in a luminal ground state by formation of clonal, acinus-like colonies expressing luminal K19 and mucin 1 (MUC1) in a correctly polarized manner (Fig. 5A and 5B left). The epithelial spheres were essentially free of myoepithelial differentiation (Fig. 5B left). To gauge for bipotency the medium was shifted to TGFβR2i-1 medium to facilitate multi-lineage propagation. Accordingly, within 72 h K14^+^ cells emerged from the rim of about one third of the K19^+^/K14^−^ spheres to indicate de novo formation of myoepithelial-like cells (Fig. 5B right and C).

**Fig. 6:**
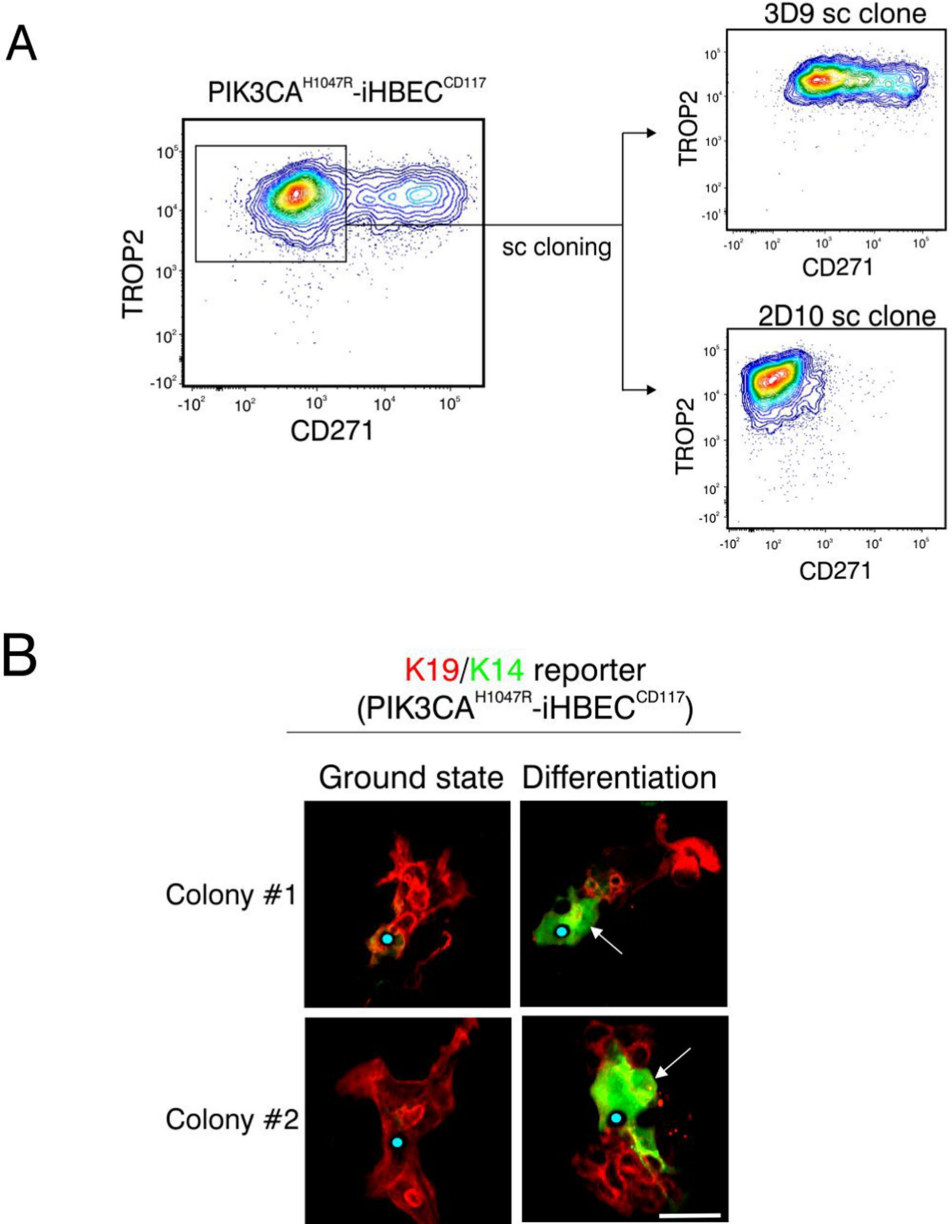
Single cell cloning and lineage tracing reveal that both lineage-restricted and bipotent progenitors are present within the PIK3CA^H1047R^-iHBEC^CD117^ cell line. **A** Representative FACS plots illustrating the gating strategy for the generation of single cell (sc) clones from the luminal progenitor cell line. TROP2^+^/CD271^−^ cells were sorted (left) as single cells and expanded. Note the presence of both bipotent (3D9) and lineage restricted (2D10) clones. **B** Multicolor live cell imaging of PIK3CA^H1047R^-iHBEC^CD117^ progenitor cells at clonal density expressing a K19-tagged miRFP670 (red) and K14 promoter-driven RFP reporter (green). Colonies were traced during ground state culture (left) and after differentiation (right). Two representative colonies are shown as a demonstration of K14^+^ cells emerging from K19^+^ luminal cells with blue dots indicating the same position on the slides at different time points. Scale bar = 50 µm

The observed lineage-related keratin expression in PIK3CA^H1047R^-iHBEC^CD117^ in multi-lineage conditions was accompanied by a typical surface marker expression of TROP2/CD271 similar to the one used to enrich uncultured cells (Fig. 6A). To confirm the cell states based on these surface markers, sorted TROP2^+^/CD271^−^ and TROP2^+^/CD271^+^ PIK3CA^H1047R^-iHBEC^CD117^ cells were subjected to bulk RNA-seq analysis. We confirmed that CD271 indeed faithfully identified myoepithelial cells and that PIK3CA^H1047R^-iHBEC^CD117^ remarkably exhibited elaborate luminal epithelial and myoepithelial differentiation programs (Additional file 1: Fig. S4A). To substantiate bipotency also among clonal colonies, we performed single-cell cloning of parental cells from the TROP2^+^/CD271^−^ gate and re-analyzed the clones by FACS after four weeks of expansion. We found that seven out of thirteen single cell-derived clones exhibited bipotency, while the rest remained essentially luminal-restricted, as exemplified here by two of the clones 3D9 and 2D10, respectively (Fig. 6A). Furthermore, only the bipotent clones readily responded to a new round of switch from a luminal-restricted ground state to multi-lineage conditions in the same way as the parental PIK3CA^H1047R-^iHBEC^CD117^ progenitors (Additional file 1: Fig. S4B).

To verify that the observed bipotency reflected a true transition from luminal epithelial to myoepithelial-like lineage, we constructed lineage-specific K19 and K14 reporters in PIK3CA^H1047R^-iHBEC^CD117^ cells. Thus, we first generated a K19 fluorescent reporter by use of CRISPR/Cas9 gene editing, and fused miRFP670 into the 3’ end of endogenous *KRT19* genomic DNA. These cells were further transduced with a K14 promoter-RFP construct to monitor the differentiation capacity to the myoepithelial lineage. In this manner, it was possible by live cell imaging to follow the induction of K14 concomitantly with the dynamics of K19 expression upon switching from luminal ground state- to multi-lineage conditions. We found that K19^−^/K14^+^ cells emerged from K19^+^/K14^−^ colonies occasionally via K19^+^/K14^+^ intermediates (Fig. 6B). Stated differently, PIK3CA^H1047R^-iHBEC^CD117^ epithelial progenitors serve as a direct source of myoepithelial-like cells.

### De novo generated myoepithelial-like cells participate in physiologically relevant morphogenesis

The above observations extended and supported our and others’ previous reports indicating that myoepithelial cells may originate from luminal progenitors under experimental conditions [51–55]. Nevertheless, whether experimentally generated, oncogene-transduced myoepithelial-like cells maintain a relevant differentiation repertoire has remained an open question. In the resting human breast, myoepithelial cells form a more or less continuous layer between the basement membrane and an inner layer of luminal epithelial cells, and we therefore hypothesized that a bilayered organization of luminal epithelial cells and differentiated myoepithelial cells would require 3D organization in a physiological environment.

**Fig. 7:**
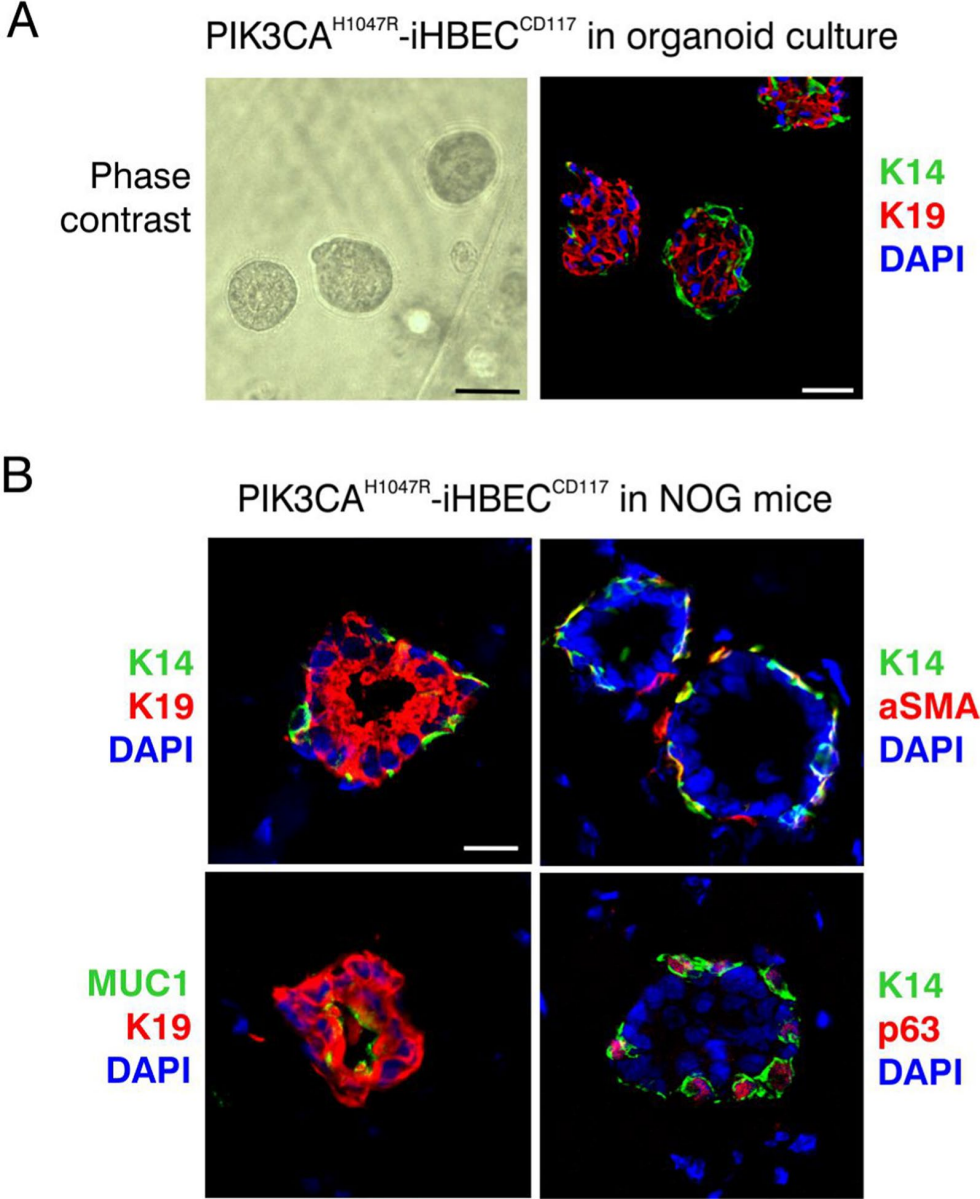
PIK3CA^H1047R^-iHBEC^CD117^ bipotent progenitors express luminal- and myoepithelial lineage programs. **A** Micrographs of luminal progenitor cells cultured in 3D in Matrigel™/collagen matrix with fibroblasts. Phase contrast micrograph (left) shows acinus-like spheres with a central lumen and at a size of approximately 50 µm after two weeks of culture. A representative multicolor imaging (right) of cryostat sections of 3D gels stained for K19 (red) and K14 (green). Note the correctly polarized position of luminal and myoepithelial-like cells. Nuclei are stained blue with DAPI. Scale bar = 50 µm. **B** Multicolor imaging of cryostat sections of PIK3CA^H1047R^-iHBEC^CD117^ transplanted to NOG mice together with fibroblasts and a Matrigel™/collagen matrix. Polarity and lumen formation are highlighted by staining of the K19^+^ luminal cells (red, left column) against basal keratin K14 and apical MUC 1 (green, left column). Myoepithelial differentiation is highlighted by staining for aSMA and p63 (red, right column) against K14 (green, right column). Nuclei are stained with DAPI (blue). n = 12 injections. Scale bar = 20 µm

In 3D organoid culture including extracellular matrices and stromal cells, PIK3CA^H1047R^-iHBEC^CD117^ formed correctly polarized bi-layered spherical structures as shown by staining for K19 in luminal and K14 in myoepithelial-like cells (Fig. 7A). Moreover, when PIK3CA^H1047R^-iHBEC^CD117^ was xenotransplanted into NOG mice, the physiologically relevant acinus-like polarized structures were further elaborated, and an even more complete differentiation program, including the expression of α-smooth muscle actin (aSMA) and p63 emerged (Fig. 7B, Additional file 1: Fig. S5). Similar data were obtained with bipotent clone 3D9 of PIK3CA^H1047R^-iHBEC^CD117^ (Additional file 1: Fig. S6). However, we failed not only to find myoepithelial-like cells, but also to observe any relevant structures from xenotransplanted luminal-restricted clones (i.e. 2D10). Moreover, in the same setup of xenotransplantation experiments, neither iHBEC^CD117^ nor the hormone sensing luminal cell line iHBEC^ERpos^ [24, 25] were able to form any structures. Interestingly, when hormone sensing cells were further transduced with shp53 and PIK3CA^H1047R^, these PIK3CA^H1047R^-iHBEC^ERpos^ cells were able to form structures, albeit to a lesser degree, and in general, without a layer of myoepithelial-like cells compared to PIK3CA^H1047R^-iHBEC^CD117^ (data not shown). Taken together, these findings suggest that the PIK3CA^H1047R^- iHBEC^CD117^ population comprises bipotent luminal progenitors capable of providing de novo mature myoepithelial-like cells.

## Discussion

Myoepithelial cells have been linked to both tumor suppression and stem cell activity [2, 17, 56, 57]. However, the exact contribution of myoepithelial- and luminal epithelial progenitors to tumor evolution is complicated by disturbances of lineage fidelity in the course of oncogenic activation [17]. This entails a conundrum since lineage fidelity is intact under normal homeostasis conditions. In the field of mouse mammary gland bio-logy there is a general consensus that lineages are supplied only by a common basally located stem cell during development and with experimental passaging of cells to cleared fat pads [58]. In adult mice, cells are replenished by self-duplication within lineages [58]. This has been documented by use of lineage tracing [59]. Although this concept stands essentially unchallenged as of today, still, data generated by this technology are interpreted with caution not least due to the fact that rare multipotent cells potentially may escape labelling [60]. Indeed, reports of the existence of multipotent progenitors even under homeostasis conditions have surfaced also in the mouse mammary gland field [61, 62], and some even narrow down bipotency to the luminal epithelial lineage as source of myoepithelial cells [63, 64]. As far as the human breast is concerned a hierarchy similar to that of mice has been predicted based on the demonstration of a stem-like cell population after grafting of immunocompromised mice [26, 65]. Likewise, normal, un-cultured luminal progenitors have been found also to give rise to sensible morphogenesis under similar conditions, albeit at a relatively lower frequency [46, 55]. Whereas it is not possible to perform conventional lineage tracing in humans, lineage tracing based on histological identification of mitochondrial cytochrome c oxidase has led to the conclusion that the majority of clonal patches originate in progenitors endowed with bipotency [66]. Thus, multiple lines of evidence indicate that under experimental conditions, luminal progenitors are bipotent with the ability to contribute de novo generated cells to the myoepithelial lineage, but it has remained an open question whether such bipotency is operational in benign lesions possibly preceding malignancy [9]. Here, we took advantage of a TROP2/CD271/CD117 based FACS protocol and a transduction protocol of the most common breast cancer oncogenes and tumor suppressor genes, which allowed us to undertake a more detailed analysis of whether myoepithelial-like cells could be generated from non-malignant luminal progenitors. Our findings suggest that human breast luminal progenitors, but not hormone sensing luminal cells, in spite of transduction with hTERT, shp16, shp53 and PIK3CA^H1047R^ are exclusively capable of de novo generation of myoepithelial-like cells, and in an in vivo model, the resultant morphogenesis is reminiscent of that of the double-layered structure of the normal breast epithelium. This response also differs from that of two recently described myoepithelial-derived progenitors subjected to similar oncogenic insults, which either form abortive structures or biphasic, hyperplastic lesions [17].

While these findings show that luminal progenitors contribute basally located myoepithelial-like cells, the above experiments leave several questions unanswered about the myoepithelial cells generated this way. It is possible that luminal bipotency is a default reaction to cellular de-stabilization like that inflicted by for instance oncogenic activation or micro-environmental imbalances [40, 51, 52, 54, 55, 58, 67, 68]. Nevertheless, luminal-derived, oncogene-activated myoepithelial-like cells may not be different from “naïve” myoepithelial cells, since in mice they have been shown to participate in correctly polarized, benign adenomyoepitheliomas, and when part of the malignant clone itself in human breast cancer, they position correctly and basally in intraductal lesions [66–68].

In terms of cell-of-origin, live-cell imaging occasionally revealed K19/K14 double positive cells prior to conversion into K19^−^/K14^+^ cells. We and others have provided ample evidence that double positive cells are endowed with bipotency [1, 46, 53, 69–72]. However, in mice bipotent luminal cells remain interpreted as a product of basal stem cells, and as such, they are currently referred to as transit- or lineage primed cells [58, 64]. In line with this, recent experiments using single cell profiling to define cellular identities and differentiation trajectories have revealed clusters with an intermediary position across luminal and myoepithelial identities [3–5, 48, 73]. While the exact order of events in the human breast lineage development remains to be understood, the present study, nevertheless, suggests that mature myoepithelial cells may originate from double-positive progenitors.

At present, intermediary cells are likely to localize in one of the stem cell zones defined by the anatomical division of the human breast into ducts and lobules [7, 26, 53] and to some extent paralleled in the mammary glands of mice [62]. To search for markers of luminal progenitor cells applicable to scRNA-seq datasets for ducts and lobules, respectively [7], we used one of the most comprehensive catalogues of human breast epithelial cell states [43]. We identified 22 genes represented in luminal progenitor and mature cells that generally showed higher expression in ducts and TDLUs, respectively. Among them, progenitor marker K15 has been previously identified as preferentially expressed in ductal progenitors [7, 41]. Moreover, within the matched genes we were further able to optimize multicolor immunofluorescence staining for four proteins: CALML5, K23, ALDH1A3 and OLFM4. CALML5 is a calcium binding protein expressed in the upper layers of stratified epithelia and is known for its tumor suppressive properties [74, 75]. In the human breast, pseudotime trajectory analysis of scRNA-seq data has mapped *CALML5* to differentiating luminal progenitor cells immediately prior to maturation [6]. In addition, using spatial transcriptomics *CALML5* has been reported as one of the differentially expressed genes with higher expression in lobules than ducts [41]. Here, we confirmed this difference at the protein level as CALML5 staining was most intense in a subset of lobuli at the most peripheral acini indicating differences within lobuli with respect to state of differentiation. K23 is known to be highly expressed in colon cancer and triple negative breast cancer where it promotes proliferation of tumor cells [76]. Monocle-generated pseudotemporal differentiation ordering of scRNA-seq data from human breast tissue has placed *KRT23* expressing cells in a bipotential root cluster [48]. We here found K23 staining complementary to CALML5 in luminal progenitors of ducts and terminal ducts, and thus very much resembling the previously reported pattern of K15 expression [7]. ALDH1A3 is a member of a family of ALDH enzymes critical in retinoic acid production [77]. In the human breast it shows a transient upregulation within epithelial progenitors at the point of commitment to the luminal lineage [78]. Similar to K23, ALDH1A3 is expressed in the luminal progenitors of ducts and terminal ducts, essentially mutually exclusive to CALML5. OLFM4 is a secreted glycoprotein belonging to the OLFM family and a marker of intestinal stem cells which is increased in the early stages of tumor initiation [79]. In line with this, it is expressed at a higher level in in situ breast cancer than in overtly invasive tumors [80]. Taken together, our findings establish a key function for luminal progenitors in ducts as a potential source of myoepithelial cells in proliferative lesions with oncogene activated plasticity.

The present findings do not exclude the possibility that lineage infidelity goes both ways, which means that luminal progenitor derived myoepithelial-like cells are also capable of generating luminal epithelial cells in a reciprocal manner. Based on our previous findings that myoepithelial cells in the terminal duct region become bipotent by the same combination of oncogenes as in the present study, and the finding that PIK3CA^H1047R^ induces alveogenic mimicry prior to tumor formation, it is indeed possible that lineages are connected in pre-malignant lesions [17, 81]. Thus, as also argued by others [82], further studies of PIK3CA^H1047R^ mutated clone dynamics in benign breast lesions implicating myoepithelial cells are needed. In meanwhile, we suggest that the condition of oncogene activated breast progenitors is referred to as being metastable. On that note, we believe that further insight into the luminal-basal spectrum of breast epithelial cell states can help address the contemporary key question of how myoepithelial integrity, in spite of its hitherto widely accepted tumor suppressive role, may causally relate to tumor recurrence.

## Supplementary Information

Additional file 1: Supplementary figures.

## Data Availability

The scRNA-seq datasets are available in the European Genome-Phenome Archive (EGA) under Study IDs EGAS00001005963 and EGAS00001005933 for primary tissue, and EGAS50000000505 for iHBEC^CD117^ cell line along with bulk RNA-seq dataset.

## Abbreviations

ALDH1A3: Aldehyde Dehydrogenase 1 family member A3
aSMA: α-Smooth muscle actin
CD26: Dipeptidyl peptidase-4, DPP4
CD117: C-kit
CD105: Endoglin, ENG
CD166: Activated leukocyte cell adhesion molecule, ALCAM
CD271: Low affinity nerve growth factor receptor, NGFR
CALML5: Calmodulin like 5
CRISPR: Clustered Regularly Interspaced Short Palindromic Repeats
Cas9: CRISPR associated protein 9
DMEM/F-12: Dulbecco's Modified Eagle Medium/Nutrient Mixture F-12
DPT: Diffusion pseudotime
ETD: Extralobular terminal ducts
FACS: Fluorescence activated cell soring
FPKM: Fragments per kilobase million
GS: Glycine/serine
hTERT: Human telomerase reverse transcriptase
K: Keratin
LP: Luminal progenitor
ML: Mature luminal
MUC1: Mucin 1
myCAF: Myofibroblast-like cancer-associated fibroblast
OLFM4: Olfactomedin 4
PAM: Protospacer adjacent motifs
PIK3CA: Phosphatidylinositol-4,5-Bisphosphate 3-Kinase Catalytic Subunit Alpha
scRNA-seq: Single cell RNA-sequencing
TDLUs: Terminal duct lobular units
TROP2: Trophoblast cell surface antigen 2
UMAP: Unifold Manifold Approximation and Projection

## References

[1] Petersen OW, Polyak K. Stem cells in the human breast. Cold Spring Harb Perspect Biol. 2010;2(5):a003160. 20452965 10.1101/cshperspect.a003160 PMC2857168

[2] Gudjonsson T, Adriance MC, Sternlicht MD, Petersen OW, Bissell MJ. Myoepithelial cells: their origin and function in breast morphogenesis and neoplasia. J Mammary Gland Biol Neoplasia. 2005;10(3):261–72. 16807805 10.1007/s10911-005-9586-4 PMC2798159

[3] Chen W, Morabito SJ, Kessenbrock K, Enver T, Meyer KB, Teschendorff AE. Single-cell landscape in mammary epithelium reveals bipotent-like cells associated with breast cancer risk and outcome. Commun Biol. 2019;2:306. 31428694 10.1038/s42003-019-0554-8 PMC6689007

[4] Pal B, Chen Y, Vaillant F, Capaldo BD, Joyce R, Song X, Bryant VL, Penington JS, Di Stefano L, Tubau Ribera N, et al. A single-cell RNA expression atlas of normal, preneoplastic and tumorigenic states in the human breast. EMBO J. 2021;40(11):e107333. 33950524 10.15252/embj.2020107333 PMC8167363

[5] Henry S, Trousdell MC, Cyrill SL, Zhao Y, Feigman MJ, Bouhuis JM, Aylard DA, Siepel A, Dos Santos CO. Characterization of gene expression signatures for the identification of cellular heterogeneity in the developing mammary gland. J Mammary Gland Biol Neoplasia. 2021;26(1):43–66. 33988830 10.1007/s10911-021-09486-3 PMC8217035

[6] Gleeson JP, Chaudhary N, Fein KC, Doerfler R, Hredzak-Showalter P, Whitehead KA. Profiling of mature-stage human breast milk cells identifies six unique lactocyte subpopulations. Sci Adv. 2022;8(26):eabm6865. 35767604 10.1126/sciadv.abm6865 PMC9242445

[7] Kohler KT, Goldhammer N, Demharter S, Pfisterer U, Khodosevich K, Rønnov-Jessen L, Petersen OW, Villadsen R, Kim J. Ductal keratin 15(+) luminal progenitors in normal breast exhibit a basal-like breast cancer transcriptomic signature. NPJ Breast Cancer. 2022;8(1):81. 35821504 10.1038/s41523-022-00444-8 PMC9276673

[8] Centonze A, Lin S, Tika E, Sifrim A, Fioramonti M, Malfait M, Song Y, Wuidart A, Van Herck J, Dannau A, et al. Heterotypic cell-cell communication regulates glandular stem cell multipotency. Nature. 2020;584(7822):608–13. 32848220 10.1038/s41586-020-2632-y PMC7116172

[9] Hu M, Yao J, Carroll DK, Weremowicz S, Chen H, Carrasco D, Richardson A, Violette S, Nikolskaya T, Nikolsky Y, et al. Regulation of in situ to invasive breast carcinoma transition. Cancer Cell. 2008;13(5):394–406. 18455123 10.1016/j.ccr.2008.03.007 PMC3705908

[10] Sternlicht MD, Barsky SH. The myoepithelial defense: a host defense against cancer. Med Hypotheses. 1997;48(1):37–46. 9049988 10.1016/s0306-9877(97)90022-0

[11] Sirka OK, Shamir ER, Ewald AJ. Myoepithelial cells are a dynamic barrier to epithelial dissemination. J Cell Biol. 2018;217(10):3368–81. 30061105 10.1083/jcb.201802144 PMC6168248

[12] Risom T, Glass DR, Averbukh I, Liu CC, Baranski A, Kagel A, McCaffrey EF, Greenwald NF, Rivero-Gutierrez B, Strand SH, et al. Transition to invasive breast cancer is associated with progressive changes in the structure and composition of tumor stroma. Cell. 2022;185(2):299. 35063072 10.1016/j.cell.2021.12.023 PMC8792442

[13] Inayatullah M, Mahesh A, Turnbull AK, Dixon JM, Natrajan R, Tiwari VK. Basal-epithelial subpopulations underlie and predict chemotherapy resistance in triple-negative breast cancer. EMBO Mol Med. 2024;16(4):823–53. 38480932 10.1038/s44321-024-00050-0 PMC11018633

[14] Lim E, Vaillant F, Wu D, Forrest NC, Pal B, Hart AH, Asselin-Labat ML, Gyorki DE, Ward T, Partanen A, et al. Aberrant luminal progenitors as the candidate target population for basal tumor development in BRCA1 mutation carriers. Nat Med. 2009;15(8):907–13. 19648928 10.1038/nm.2000

[15] Molyneux G, Geyer FC, Magnay FA, McCarthy A, Kendrick H, Natrajan R, Mackay A, Grigoriadis A, Tutt A, Ashworth A, et al. BRCA1 basal-like breast cancers originate from luminal epithelial progenitors and not from basal stem cells. Cell Stem Cell. 2010;7(3):403–17. 20804975 10.1016/j.stem.2010.07.010

[16] Okkenhaug K, Roychoudhuri R. Oncogenic PI3Kalpha promotes multipotency in breast epithelial cells. Sci Signal. 2015;8(401):pe3. 26535006 10.1126/scisignal.aad5856 PMC4637986

[17] Goldhammer N, Kim J, Villadsen R, Rønnov-Jessen L, Petersen OW. Myoepithelial progenitors as founder cells of hyperplastic human breast lesions upon PIK3CA transformation. Commun Biol. 2022;5(1):219. 35273332 10.1038/s42003-022-03161-x PMC8913783

[18] Rønnov-Jessen L, Petersen OW. Induction of alpha-smooth muscle actin by transforming growth factor-beta 1 in quiescent human breast gland fibroblasts. Implications for myofibroblast generation in breast neoplasia. Lab Invest. 1993;68(6):696–707. 8515656

[19] Stampfer M, Hallowes RC, Hackett AJ. Growth of normal human mammary cells in culture. In Vitro. 1980;16(5):415–25. 6993343 10.1007/BF02618365

[20] Hayer A, Shao L, Chung M, Joubert LM, Yang HW, Tsai FC, Bisaria A, Betzig E, Meyer T. Engulfed cadherin fingers are polarized junctional structures between collectively migrating endothelial cells. Nat Cell Biol. 2016;18(12):1311–23. 27842057 10.1038/ncb3438 PMC6159904

[21] Szulc J, Wiznerowicz M, Sauvain MO, Trono D, Aebischer P. A versatile tool for conditional gene expression and knockdown. Nat Methods. 2006;3(2):109–16. 16432520 10.1038/nmeth846

[22] Ng PK, Li J, Jeong KJ, Shao S, Chen H, Tsang YH, Sengupta S, Wang Z, Bhavana VH, Tran R, et al. Systematic functional annotation of somatic mutations in cancer. Cancer Cell. 2018;33(3):450. 29533785 10.1016/j.ccell.2018.01.021 PMC5926201

[23] Fridriksdottir AJ, Kim J, Villadsen R, Klitgaard MC, Hopkinson BM, Petersen OW, Rønnov-Jessen L. Propagation of oestrogen receptor-positive and oestrogen-responsive normal human breast cells in culture. Nat Commun. 2015;6:8786. 26564780 10.1038/ncomms9786 PMC4660059

[24] Hopkinson BM, Klitgaard MC, Petersen OW, Villadsen R, Rønnov-Jessen L, Kim J. Establishment of a normal-derived estrogen receptor-positive cell line comparable to the prevailing human breast cancer subtype. Oncotarget. 2017;8(6):10580–93. 28076334 10.18632/oncotarget.14554 PMC5354682

[25] Rønnov-Jessen L, Kim J, Goldhammer N, Klitgaard MC, Smicius M, Bechmann MB, Villadsen R, Petersen OW. Desensitization of human breast progenitors by a transient exposure to pregnancy levels of estrogen. Sci Rep. 2021;11(1):17232. 34446796 10.1038/s41598-021-96785-8 PMC8390656

[26] Fridriksdottir AJ, Villadsen R, Morsing M, Klitgaard MC, Kim J, Petersen OW, Rønnov-Jessen L. Proof of region-specific multipotent progenitors in human breast epithelia. Proc Natl Acad Sci U S A. 2017;114(47):E10102–11. 29109259 10.1073/pnas.1714063114 PMC5703323

[27] Morsing M, Klitgaard MC, Jafari A, Villadsen R, Kassem M, Petersen OW, Rønnov-Jessen L. Evidence of two distinct functionally specialized fibroblast lineages in breast stroma. Breast Cancer Res. 2016;18(1):108. 27809866 10.1186/s13058-016-0769-2 PMC5093959

[28] Morsing M, Kim J, Villadsen R, Goldhammer N, Jafari A, Kassem M, Petersen OW, Rønnov-Jessen L. Fibroblasts direct differentiation of human breast epithelial progenitors. Breast Cancer Res. 2020;22(1):102. 32993755 10.1186/s13058-020-01344-0 PMC7526135

[29] Bagger MM, Sjolund J, Kim J, Kohler KT, Villadsen R, Jafari A, Kassem M, Pietras K, Rønnov-Jessen L, Petersen OW. Evidence of steady-state fibroblast subtypes in the normal human breast as cells-of-origin for perturbed-state fibroblasts in breast cancer. Breast Cancer Res. 2024;26(1):11. 38229104 10.1186/s13058-024-01763-3 PMC10790388

[30] Love MI, Huber W, Anders S. Moderated estimation of fold change and dispersion for RNA-seq data with DESeq2. Genome Biol. 2014;15(12):550. 25516281 10.1186/s13059-014-0550-8 PMC4302049

[31] Stuart T, Butler A, Hoffman P, Hafemeister C, Papalexi E, Mauck WM 3rd, Hao Y, Stoeckius M, Smibert P, Satija R. Comprehensive integration of single-cell data. Cell. 2019;177(7):1888–902. 31178118 10.1016/j.cell.2019.05.031 PMC6687398

[32] Coifman RR, Lafon S, Lee AB, Maggioni M, Nadler B, Warner F, Zucker SW. Geometric diffusions as a tool for harmonic analysis and structure definition of data: diffusion maps. Proc Natl Acad Sci U S A. 2005;102(21):7426–31. 15899970 10.1073/pnas.0500334102 PMC1140422

[33] Angerer P, Haghverdi L, Buttner M, Theis FJ, Marr C, Buettner F. destiny: diffusion maps for large-scale single-cell data in R. Bioinformatics. 2016;32(8):1241–3. 26668002 10.1093/bioinformatics/btv715

[34] Haghverdi L, Buettner F, Theis FJ. Diffusion maps for high-dimensional single-cell analysis of differentiation data. Bioinformatics. 2015;31(18):2989–98. 26002886 10.1093/bioinformatics/btv325

[35] Haghverdi L, Buttner M, Wolf FA, Buettner F, Theis FJ. Diffusion pseudotime robustly reconstructs lineage branching. Nat Methods. 2016;13(10):845–8. 27571553 10.1038/nmeth.3971

[36] Goldhammer N, Kim J, Timmermans-Wielenga V, Petersen OW. Characterization of organoid cultured human breast cancer. Breast Cancer Res. 2019;21(1):141. 31829259 10.1186/s13058-019-1233-x PMC6907265

[37] Isberg OG, Kim J, Fridriksdottir AJ, Morsing M, Timmermans-Wielenga V, Rønnov-Jessen L, Petersen OW, Villadsen R. A CD146 FACS protocol enriches for luminal keratin 14/19 double positive human breast progenitors. Sci Rep. 2019;9(1):14843. 31619692 10.1038/s41598-019-50903-9 PMC6795797

[38] Cancer Genome Atlas N: Comprehensive molecular portraits of human breast tumours. *Nature* 2012, 490(7418):61–70 10.1038/nature11412 PMC3465532 23000897

[39] Chakrabarty A, Surendran S, Bhola NE, Mishra VS, Wani TH, Baghel KS, Arteaga CL, Garg R, Chowdhury G. The H1047R PIK3CA oncogene induces a senescence-like state, pleiotropy and acute HSP90 dependency in HER2+ mammary epithelial cells. Carcinogenesis. 2019;40(10):1179–90. 31219154 10.1093/carcin/bgz118

[40] Nguyen QH, Pervolarakis N, Blake K, Ma D, Davis RT, James N, Phung AT, Willey E, Kumar R, Jabart E, et al. Profiling human breast epithelial cells using single cell RNA sequencing identifies cell diversity. Nat Commun. 2018;9(1):2028. 29795293 10.1038/s41467-018-04334-1 PMC5966421

[41] Kumar T, Nee K, Wei R, He S, Nguyen QH, Bai S, Blake K, Pein M, Gong Y, Sei E, et al. A spatially resolved single-cell genomic atlas of the adult human breast. Nature. 2023;620(7972):181–91. 37380767 10.1038/s41586-023-06252-9 PMC11443819

[42] Reed AD, Pensa S, Steif A, Stenning J, Kunz DJ, Porter LJ, Hua K, He P, Twigger AJ, Siu AJQ, et al. A single-cell atlas enables mapping of homeostatic cellular shifts in the adult human breast. Nat Genet. 2024;56(4):652–62. 38548988 10.1038/s41588-024-01688-9 PMC11018528

[43] Bhat-Nakshatri P, Gao H, Sheng L, McGuire PC, Xuei X, Wan J, Liu Y, Althouse SK, Colter A, Sandusky G, et al. A single-cell atlas of the healthy breast tissues reveals clinically relevant clusters of breast epithelial cells. Cell Rep Med. 2021;2(3):100219. 33763657 10.1016/j.xcrm.2021.100219 PMC7974552

[44] Gray GK, Li CM, Rosenbluth JM, Selfors LM, Girnius N, Lin JR, Schackmann RCJ, Goh WL, Moore K, Shapiro HK, et al. A human breast atlas integrating single-cell proteomics and transcriptomics. Dev Cell. 2022;57(11):1400–20. 35617956 10.1016/j.devcel.2022.05.003 PMC9202341

[45] Grigoriadis A, Mackay A, Reis-Filho JS, Steele D, Iseli C, Stevenson BJ, Jongeneel CV, Valgeirsson H, Fenwick K, Iravani M, et al. Establishment of the epithelial-specific transcriptome of normal and malignant human breast cells based on MPSS and array expression data. Breast Cancer Res. 2006;8(5):R56. 17014703 10.1186/bcr1604 PMC1779497

[46] Keller PJ, Arendt LM, Skibinski A, Logvinenko T, Klebba I, Dong S, Smith AE, Prat A, Perou CM, Gilmore H, et al. Defining the cellular precursors to human breast cancer. Proc Natl Acad Sci U S A. 2012;109(8):2772–7. 21940501 10.1073/pnas.1017626108 PMC3286919

[47] Thi K, Del Toro K, Licon-Munoz Y, Sayaman RW, Hines WC. Comprehensive identification, isolation, and culture of human breast cell types. J Biol Chem. 2024;300(10):107637. 39122004 10.1016/j.jbc.2024.107637 PMC11459906

[48] Peng S, Hebert LL, Eschbacher JM, Kim S: Single-cell RNA sequencing of a postmenopausal normal breast tissue identifies multiple cell types that contribute to breast cancer. *Cancers (Basel)* 2020, 12(12). 10.3390/cancers12123639 PMC7761899 33291647

[49] Murrow LM, Weber RJ, Caruso JA, McGinnis CS, Phong K, Gascard P, Rabadam G, Borowsky AD, Desai TA, Thomson M, et al. Mapping hormone-regulated cell-cell interaction networks in the human breast at single-cell resolution. Cell Syst. 2022;13(8):644. 35863345 10.1016/j.cels.2022.06.005 PMC9590200

[50] Twigger AJ, Engelbrecht LK, Bach K, Schultz-Pernice I, Pensa S, Stenning J, Petricca S, Scheel CH, Khaled WT. Transcriptional changes in the mammary gland during lactation revealed by single cell sequencing of cells from human milk. Nat Commun. 2022;13(1):562. 35091553 10.1038/s41467-021-27895-0 PMC8799659

[51] Petersen OW, van Deurs B. Growth factor control of myoepithelial-cell differentiation in cultures of human mammary gland. Differentiation. 1988;39(3):197–215. 2468550 10.1111/j.1432-0436.1988.tb00094.x

[52] Pechoux C, Gudjonsson T, Ronnov-Jessen L, Bissell MJ, Petersen OW. Human mammary luminal epithelial cells contain progenitors to myoepithelial cells. Dev Biol. 1999;206(1):88–99. 9918697 10.1006/dbio.1998.9133

[53] Villadsen R, Fridriksdottir AJ, Rønnov-Jessen L, Gudjonsson T, Rank F, LaBarge MA, Bissell MJ, Petersen OW. Evidence for a stem cell hierarchy in the adult human breast. J Cell Biol. 2007;177(1):87–101. 17420292 10.1083/jcb.200611114 PMC2064114

[54] Keller PJ, Lin AF, Arendt LM, Klebba I, Jones AD, Rudnick JA, DiMeo TA, Gilmore H, Jefferson DM, Graham RA, et al. Mapping the cellular and molecular heterogeneity of normal and malignant breast tissues and cultured cell lines. Breast Cancer Res. 2010;12(5):R87. 20964822 10.1186/bcr2755 PMC3096980

[55] Shehata M, Teschendorff A, Sharp G, Novcic N, Russell IA, Avril S, Prater M, Eirew P, Caldas C, Watson CJ, et al. Phenotypic and functional characterisation of the luminal cell hierarchy of the mammary gland. Breast Cancer Res. 2012;14(5):R134. 23088371 10.1186/bcr3334 PMC4053112

[56] Adriance MC, Inman JL, Petersen OW, Bissell MJ. Myoepithelial cells: good fences make good neighbors. Breast Cancer Res. 2005;7(5):190–7. 16168137 10.1186/bcr1286 PMC1242144

[57] Prater MD, Petit V, Alasdair Russell I, Giraddi RR, Shehata M, Menon S, Schulte R, Kalajzic I, Rath N, Olson MF, et al. Mammary stem cells have myoepithelial cell properties. Nat Cell Biol. 2014;16(10):942–50. 25173976 10.1038/ncb3025 PMC4183554

[58] Fu NY, Nolan E, Lindeman GJ, Visvader JE. Stem cells and the differentiation hierarchy in mammary gland development. Physiol Rev. 2020;100(2):489–523. 31539305 10.1152/physrev.00040.2018

[59] Van Keymeulen A, Rocha AS, Ousset M, Beck B, Bouvencourt G, Rock J, Sharma N, Dekoninck S, Blanpain C. Distinct stem cells contribute to mammary gland development and maintenance. Nature. 2011;479(7372):189–93. 21983963 10.1038/nature10573

[60] Ciwinska M, Messal HA, Hristova HR, Lutz C, Bornes L, Chalkiadakis T, Harkes R, Langedijk NSM, Hutten SJ, Menezes RX, et al. Mechanisms that clear mutations drive field cancerization in mammary tissue. Nature. 2024;633(8028):198–206. 39232148 10.1038/s41586-024-07882-3 PMC11374684

[61] Chakrabarti R, Celia-Terrassa T, Kumar S, Hang X, Wei Y, Choudhury A, Hwang J, Peng J, Nixon B, Grady JJ, et al. Notch ligand Dll1 mediates cross-talk between mammary stem cells and the macrophageal niche. Science. 2018;360:6396. 10.1126/science.aan4153 PMC7881440 29773667

[62] Rios AC, Fu NY, Lindeman GJ, Visvader JE. In situ identification of bipotent stem cells in the mammary gland. Nature. 2014;506(7488):322–7. 24463516 10.1038/nature12948

[63] Bu W, Chen J, Morrison GD, Huang S, Creighton CJ, Huang J, Chamness GC, Hilsenbeck SG, Roop DR, Leavitt AD, et al. Keratin 6a marks mammary bipotential progenitor cells that can give rise to a unique tumor model resembling human normal-like breast cancer. Oncogene. 2011;30(43):4399–409. 21532625 10.1038/onc.2011.147 PMC3156856

[64] Song W, Wang R, Jiang W, Yin Q, Peng G, Yang R, Yu QC, Chen J, Li J, Cheung TH, et al. Hormones induce the formation of luminal-derived basal cells in the mammary gland. Cell Res. 2019;29(3):206–20. 30631153 10.1038/s41422-018-0137-0 PMC6460434

[65] Eirew P, Stingl J, Raouf A, Turashvili G, Aparicio S, Emerman JT, Eaves CJ. A method for quantifying normal human mammary epithelial stem cells with in vivo regenerative ability. Nat Med. 2008;14(12):1384–9. 19029987 10.1038/nm.1791

[66] Cereser B, Jansen M, Austin E, Elia G, McFarlane T, van Deurzen CH, Sieuwerts AM, Daidone MG, Tadrous PJ, Wright NA, et al. Analysis of clonal expansions through the normal and premalignant human breast epithelium reveals the presence of luminal stem cells. J Pathol. 2018;244(1):61–70. 28940516 10.1002/path.4989 PMC5765426

[67] Koren S, Reavie L, Couto JP, De Silva D, Stadler MB, Roloff T, Britschgi A, Eichlisberger T, Kohler H, Aina O, et al. PIK3CA(H1047R) induces multipotency and multi-lineage mammary tumours. Nature. 2015;525(7567):114–8. 26266975 10.1038/nature14669

[68] Van Keymeulen A, Lee MY, Ousset M, Brohee S, Rorive S, Giraddi RR, Wuidart A, Bouvencourt G, Dubois C, Salmon I, et al. Reactivation of multipotency by oncogenic PIK3CA induces breast tumour heterogeneity. Nature. 2015;525(7567):119–23. 26266985 10.1038/nature14665

[69] Britschgi A, Duss S, Kim S, Couto JP, Brinkhaus H, Koren S, De Silva D, Mertz KD, Kaup D, Varga Z, et al. The Hippo kinases LATS1 and 2 control human breast cell fate via crosstalk with ERalpha. Nature. 2017;541(7638):541–5. 28068668 10.1038/nature20829 PMC6726477

[70] Bao B, Prasad AS. Targeting CSC in a most aggressive subtype of breast cancer TNBC. Adv Exp Med Biol. 2019;1152:311–34. 31456192 10.1007/978-3-030-20301-6_17

[71] Zhao X, Malhotra GK, Lele SM, Lele MS, West WW, Eudy JD, Band H, Band V. Telomerase-immortalized human mammary stem/progenitor cells with ability to self-renew and differentiate. Proc Natl Acad Sci U S A. 2010;107(32):14146–51. 20660721 10.1073/pnas.1009030107 PMC2922525

[72] Arendt LM, Keller PJ, Skibinski A, Goncalves K, Naber SP, Buchsbaum RJ, Gilmore H, Come SE, Kuperwasser C. Anatomical localization of progenitor cells in human breast tissue reveals enrichment of uncommitted cells within immature lobules. Breast Cancer Res. 2014;16(5):453. 25315014 10.1186/s13058-014-0453-3 PMC4303132

[73] Regan JL, Smalley MJ. Integrating single-cell RNA-sequencing and functional assays to decipher mammary cell states and lineage hierarchies. NPJ Breast Cancer. 2020;6:32. 32793804 10.1038/s41523-020-00175-8 PMC7391676

[74] Sun BK, Boxer LD, Ransohoff JD, Siprashvili Z, Qu K, Lopez-Pajares V, Hollmig ST, Khavari PA. CALML5 is a ZNF750- and TINCR-induced protein that binds stratifin to regulate epidermal differentiation. Genes Dev. 2015;29(21):2225–30. 26545810 10.1101/gad.267708.115 PMC4647556

[75] Kitazawa S, Takaoka Y, Ueda Y, Kitazawa R. Identification of calmodulin-like protein 5 as tumor-suppressor gene silenced during early stage of carcinogenesis in squamous cell carcinoma of uterine cervix. Int J Cancer. 2021;149(6):1358–68. 33997976 10.1002/ijc.33687

[76] Zhou J, Qian W, Huang C, Mai C, Lai Y, Lin Z, Lai G. Combined targeting of KRT23 and NCCRP1 as a potential novel therapeutic approach for the treatment of triple-negative breast cancer. Gland Surg. 2022;11(10):1673–82. 36353580 10.21037/gs-22-486 PMC9638800

[77] McLean ME, MacLean MR, Cahill HF, Arun RP, Walker OL, Wasson MD, Fernando W, Venkatesh J, Marcato P: The expanding role of cancer stem cell marker ALDH1A3 in cancer and beyond. *Cancers (Basel)* 2023, 15(2). 10.3390/cancers15020492 PMC9857290 36672441

[78] Eirew P, Kannan N, Knapp DJ, Vaillant F, Emerman JT, Lindeman GJ, Visvader JE, Eaves CJ. Aldehyde dehydrogenase activity is a biomarker of primitive normal human mammary luminal cells. Stem Cells. 2012;30(2):344–8. 22131125 10.1002/stem.1001

[79] Guette C, Valo I, Vetillard A, Coqueret O. Olfactomedin-4 is a candidate biomarker of solid gastric, colorectal, pancreatic, head and neck, and prostate cancers. Proteomics Clin Appl. 2015;9(1–2):58–63. 25400027 10.1002/prca.201400083

[80] Valo I, Raro P, Boissard A, Maarouf A, Jezequel P, Verriele V, Campone M, Coqueret O, Guette C. OLFM4 expression in ductal carcinoma in situ and in invasive breast cancer cohorts by a SWATH-based proteomic approach. Proteomics. 2019;19(21–22):e1800446. 31318138 10.1002/pmic.201800446

[81] Langille E, Al-Zahrani KN, Ma Z, Liang M, Uuskula-Reimand L, Espin R, Teng K, Malik A, Bergholtz H, Ghamrasni SE, et al. Loss of epigenetic regulation disrupts lineage integrity, induces aberrant alveogenesis, and promotes breast cancer. Cancer Discov. 2022;12(12):2930–53. 36108220 10.1158/2159-8290.CD-21-0865 PMC9812400

[82] Mishima C, Kagara N, Ikeda JI, Morii E, Miyake T, Tanei T, Naoi Y, Shimoda M, Shimazu K, Kim SJ, et al. Mutational analysis of AKT1 and PIK3CA in intraductal papillomas of the breast with special reference to cellular components. Am J Pathol. 2018;188(5):1106–12. 29454754 10.1016/j.ajpath.2018.01.005

