# Supplementary material for: Oncogene activated human breast luminal progenitors contribute basally located myoepithelial cells
Source: Breast Cancer Res. 2024 Dec 18;26:183. doi: 10.1186/s13058-024-01939-x (PMC11656586; doi:10.1186/s13058-024-01939-x)
Supplement: Supplementary file 1 — Additional file 1: Supplementary figures. [file 13058_2024_1939_MOESM1_ESM.docx]

**
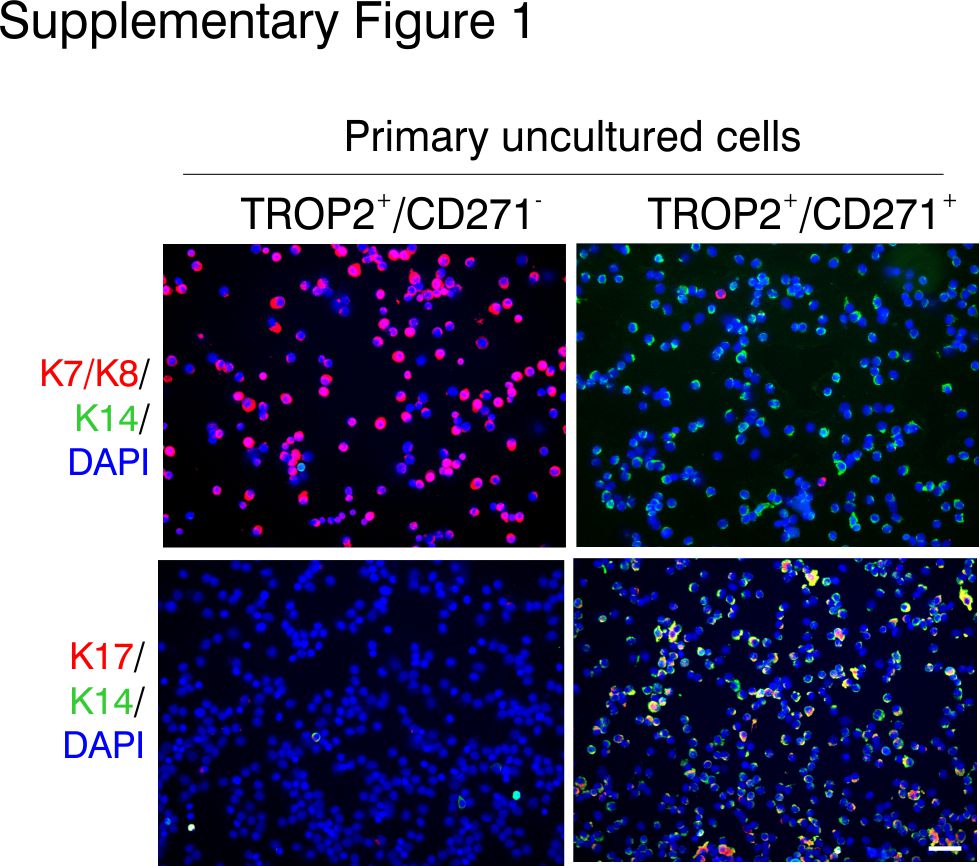
Supplementary Fig. 1** Isolation and characterization of luminal and myoepithelial cells from human breast tissue.

Multicolor imaging of uncultured, FACS sorted single cells from a TROP^+^/CD271^-^ gate and a TROP2^+^/CD271^+^ gate. Cells were smeared on glass slides and stained with CAM5.2 that recognizes K7/K8 (red) and K14 (green; upper row) and K17 (red) and K14 (green; lower row). Scale bar = 50 µm

**
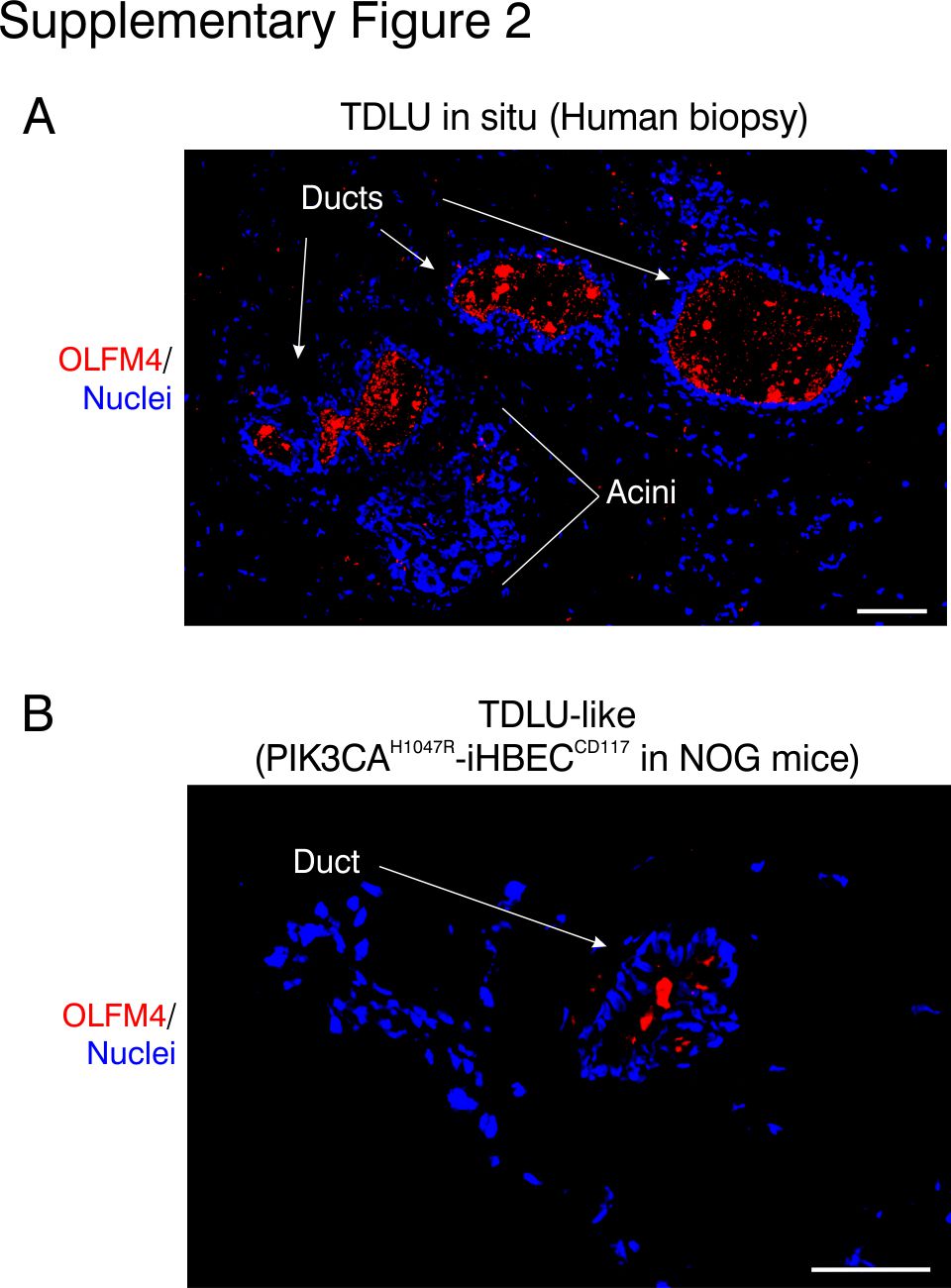
Supplementary Fig. 2** OLFM4 is highly expressed in ducts in situ and in vivo.

Multicolor imaging of paraffin sections of **A** human breast tissue, and **B** PIK3CA^H1047R^-iHBEC^CD117^ in NOG mice stained for OLFM4 (red) and DAPI staining of nuclei (blue). In breast tissue, when OLFM4 staining was detected, it was restricted to ducts (8 out of 10 biopsies). Note staining of duct-like structures in breast tissue and in some of the profiles in NOG mice. Scale bar = 100 µm (A) and 50 µm (B).

**
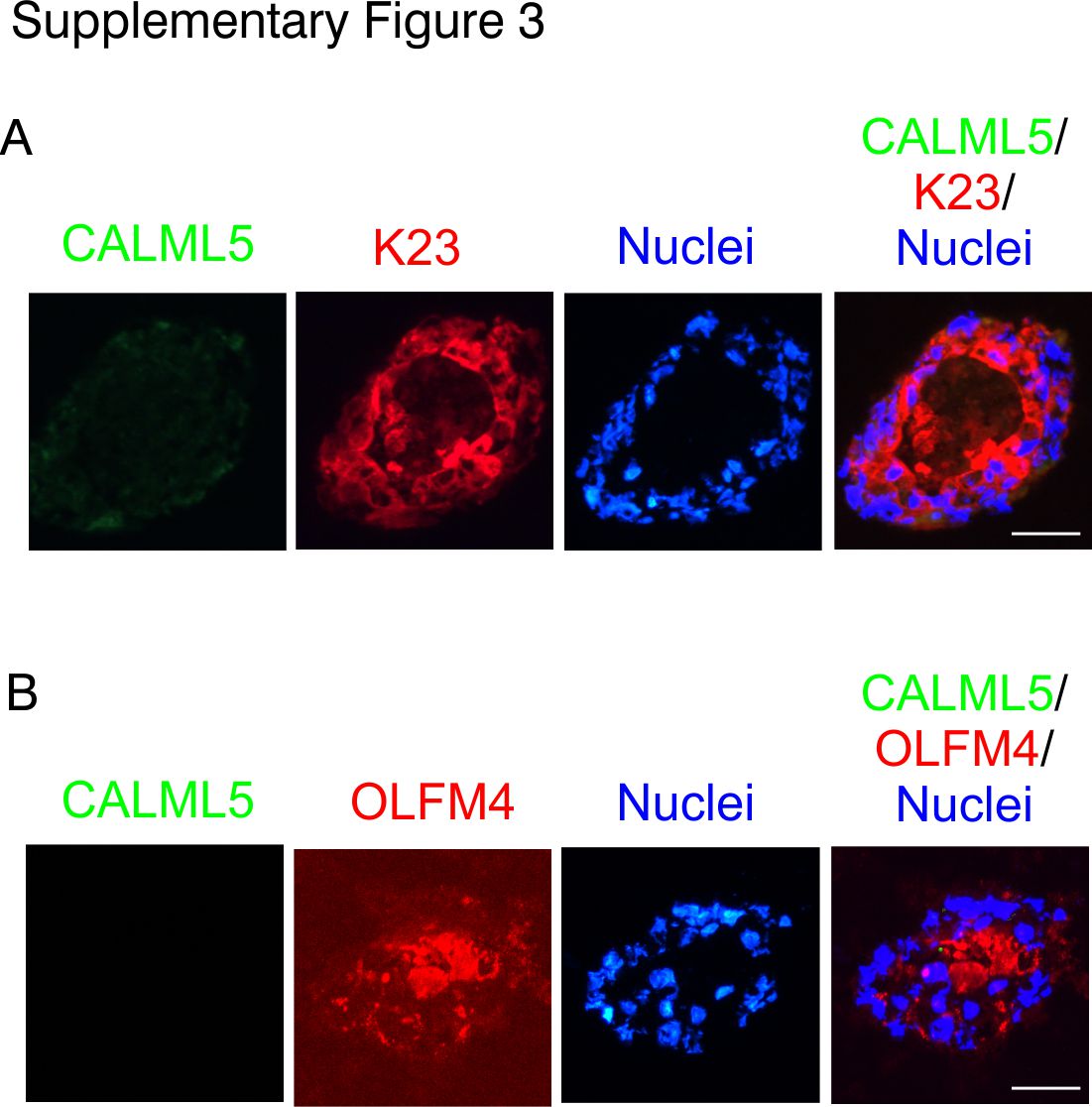
Supplementary Fig. 3** PIK3CA^H1047R^-iHBEC^CD117^ in organoid culture show expression of ductal markers K23 and OLFM4.

PIK3CA^H1047R^-iHBEC^CD117^ organoid cultures were stained for CALML5 (green) and K23 (red) (A) and CALML5 (green) and OLFM4 (red) (B). While no staining of CALML5 was observed, K23 and OLFM4 were broadly expressed. Scale bar = 50 µm.

**
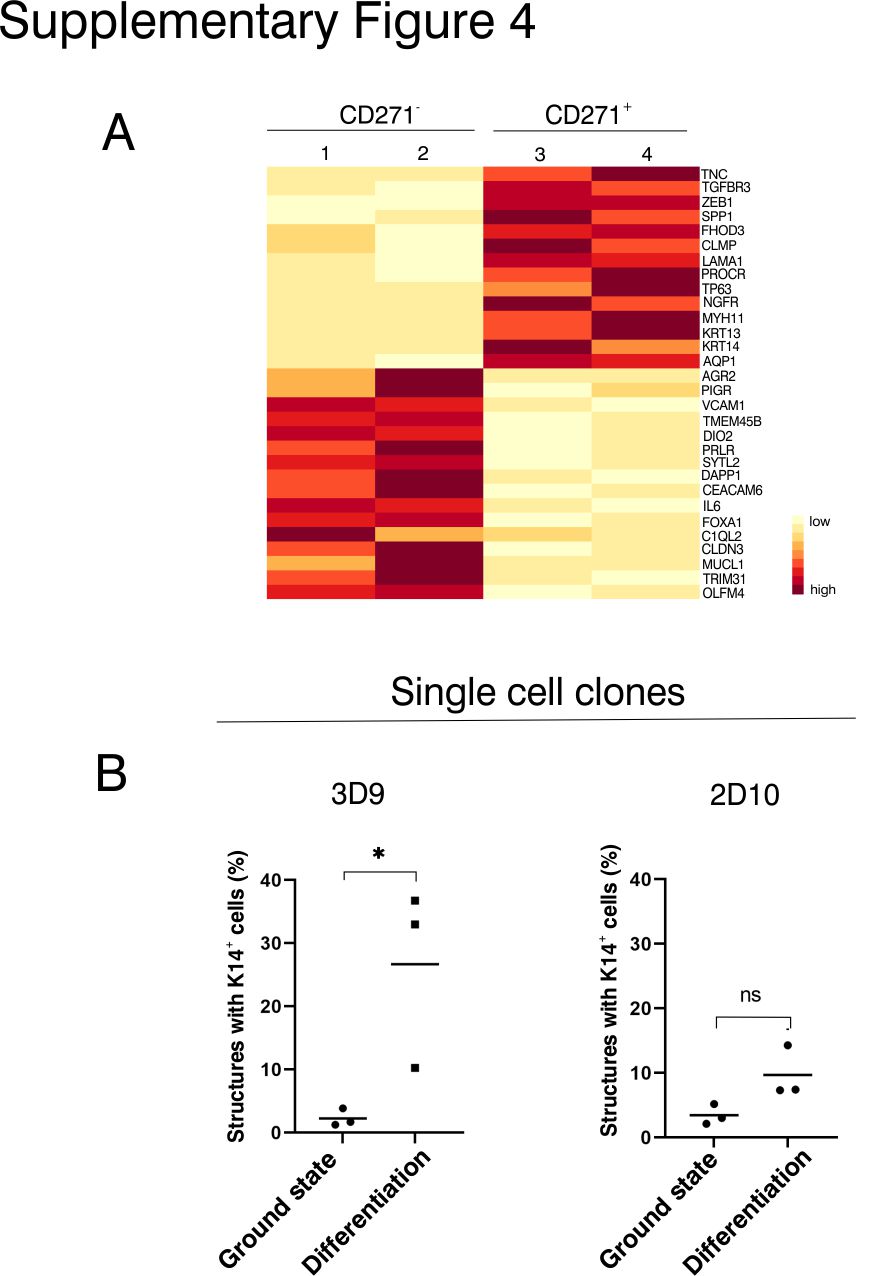
Supplementary Fig. 4** A subset of PIK3CA^H1047R^-iHBEC^CD117^ can give rise to the myoepithelial-like cells.

**A** Heatmap showing expression profiles of the luminal- and myoepithelial-like lineage in FACS sorted TROP2^+^/CD271^-^ (CD271^-^) and TROP2^+^/CD271^+^(CD271^+^) cells from the PIK3CA^H1047R^-iHBEC^CD117^ progenitor cell line. FPKM expression values are scaled across each gene, with red and yellow representing high and low relative expression, respectively (n=2 per group). While CD271^-^ cells show high expression of luminal-specific genes, myoepithelial markers are highly expressed in CD271^+^ cells. **B** Dot plots showing the frequency of structures positive for K14 in ground state culture versus differentiation conditions in three experiments with two clones. 3D9 clone is able to give rise to K14^+^ cells (left), while 2D10 is more luminal-restricted (right). *p < 0.05; ns=not significant, tested by two-tailed t test.

**
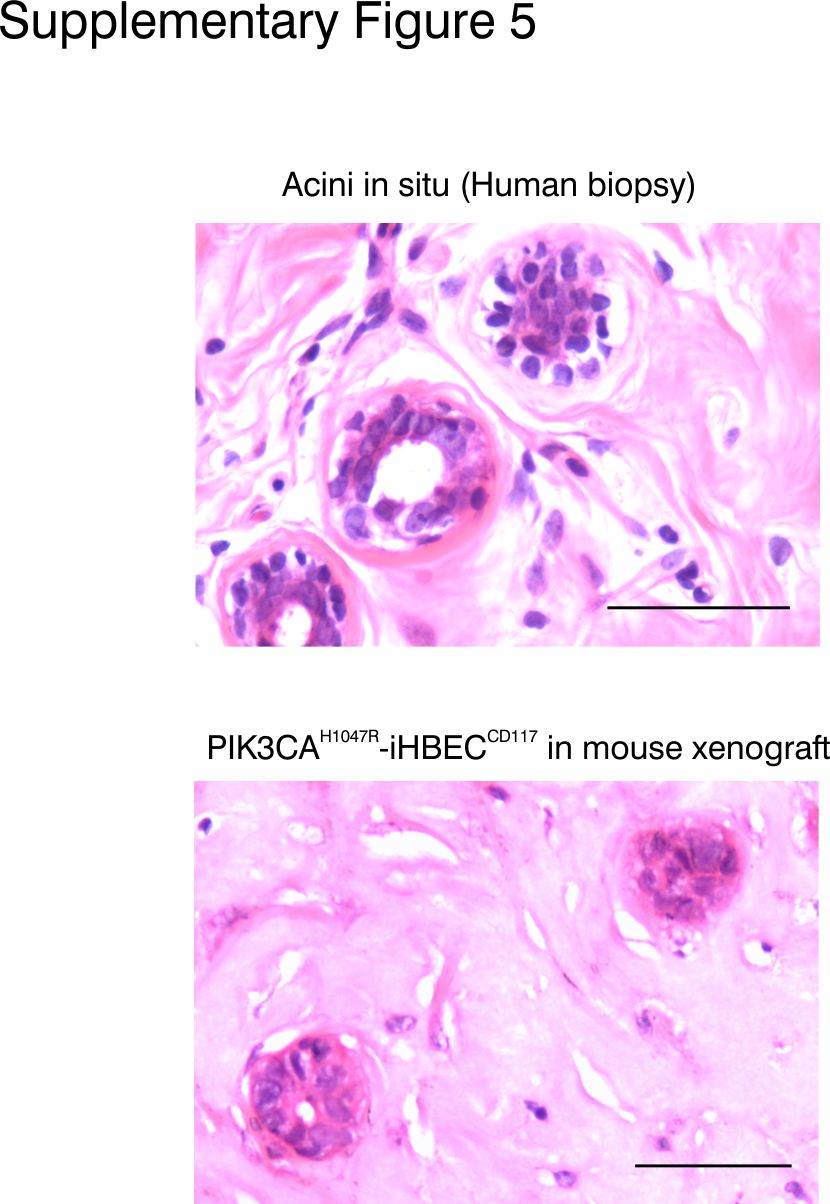
Supplementary Fig. 5** Structures from PIK3CA^H1047R^-iHBEC^CD117^ in mouse xenografts resemble acini of normal tissue.

Hematoxylin/Eosin staining of paraffin embedded samples derived from primary human breast tissue (upper) and mouse xenografts (lower). Scale bars = 50 µm.

**
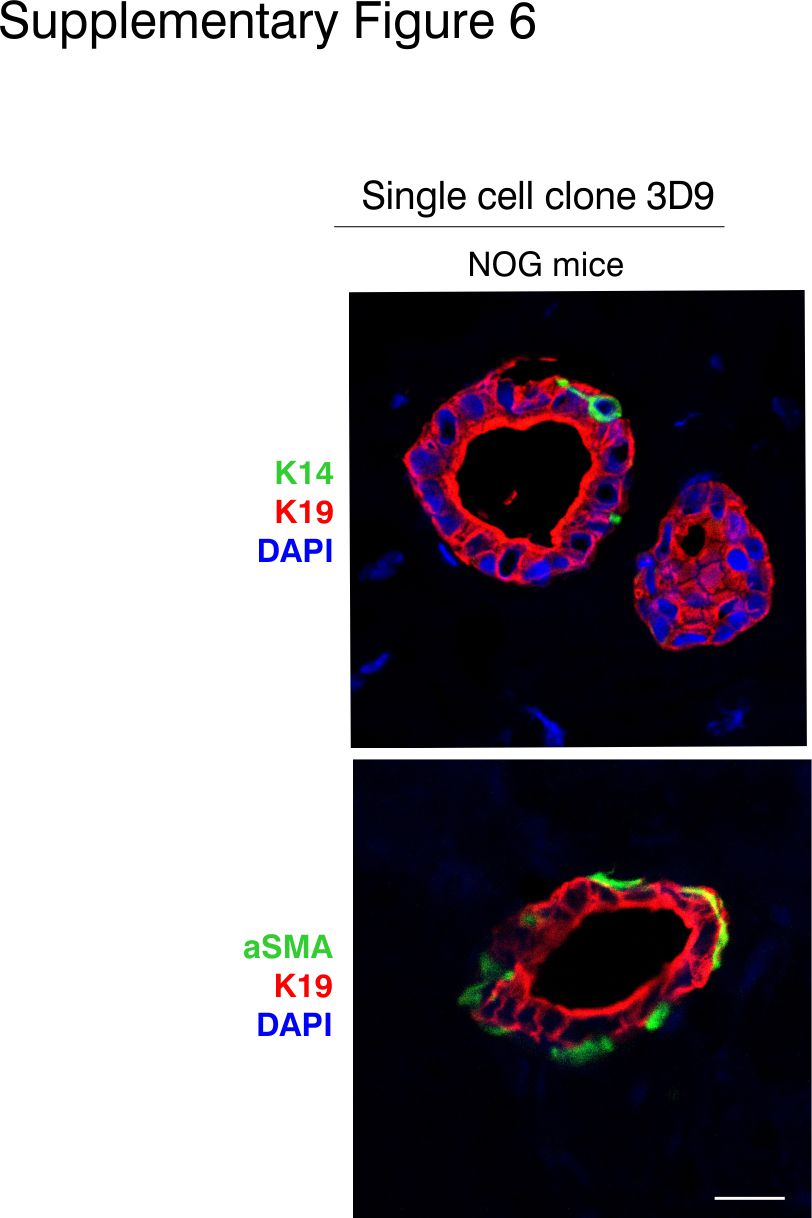
Supplementary Fig. 6** Bipotent 3D9 clone forms double layered structures in mice.

Multicolor imaging of 3D9 sc clone transplanted into NOG mice. Cryostat sections were stained for K19 (red), K14 (green, upper) and aSMA (green, lower). As with the PIK3CA^H1047R^-iHBEC^CD117^ parental progenitors, the single cell derived clone 3D9 forms double layered structures with basal K14^+^/aSMA^+^ cells, whereas 2D10 clone failed to form structures. n = 4 injections per clone. Scale bar = 20 µm.
